# The *Physcomitrella patens* Chloroplast Proteome Changes in Response to Protoplastation

**DOI:** 10.3389/fpls.2016.01661

**Published:** 2016-11-04

**Authors:** Igor Fesenko, Anna Seredina, Georgij Arapidi, Vasily Ptushenko, Anatoly Urban, Ivan Butenko, Sergey Kovalchuk, Konstantin Babalyan, Andrey Knyazev, Regina Khazigaleeva, Elena Pushkova, Nikolai Anikanov, Vadim Ivanov, Vadim M. Govorun

**Affiliations:** ^1^Laboratory of Proteomics, Shemyakin and Ovchinnikov Institute of Bioorganic Chemistry, Russian Academy of SciencesMoscow, Russia; ^2^Department of Bioenergetics, Belozersky Institute of Physico-Chemical Biology, Lomonosov Moscow State UniversityMoscow, Russia; ^3^Department of Biocatalysis, Emanuel Institute of Biochemical Physics, Russian Academy of SciencesMoscow, Russia; ^4^Laboratory of the Proteomic Analysis, Research Institute for Physico-Chemical MedicineMoscow, Russia

**Keywords:** SWATH-MS, MRM-MS, chloroplast proteome, moss *Physcomitrella patens*, label-free quantification, stress conditions

## Abstract

Plant protoplasts are widely used for genetic manipulation and functional studies in transient expression systems. However, little is known about the molecular pathways involved in a cell response to the combined stress factors resulted from protoplast generation. Plants often face more than one type of stress at a time, and how plants respond to combined stress factors is therefore of great interest. Here, we used protoplasts of the moss *Physcomitrella patens* as a model to study the effects of short-term stress on the chloroplast proteome. Using label-free comparative quantitative proteomic analysis (SWATH-MS), we quantified 479 chloroplast proteins, 219 of which showed a more than 1.4-fold change in abundance in protoplasts. We additionally quantified 1451 chloroplast proteins using emPAI. We observed degradation of a significant portion of the chloroplast proteome following the first hour of stress imposed by the protoplast isolation process. Electron-transport chain (ETC) components underwent the heaviest degradation, resulting in the decline of photosynthetic activity. We also compared the proteome changes to those in the transcriptional level of nuclear-encoded chloroplast genes. Globally, the levels of the quantified proteins and their corresponding mRNAs showed limited correlation. Genes involved in the biosynthesis of chlorophyll and components of the outer chloroplast membrane showed decreases in both transcript and protein abundance. However, proteins like dehydroascorbate reductase 1 and 2-cys peroxiredoxin B responsible for ROS detoxification increased in abundance. Further, genes such as thylakoid ascorbate peroxidase were induced at the transcriptional level but down-regulated at the proteomic level. Together, our results demonstrate that the initial chloroplast reaction to stress is due changes at the proteomic level.

## Introduction

In nature, plants are simultaneously exposed to a combination of various stress factors. Abiotic and biotic stresses result in reduction of photosynthetic activity and possibly degradation of the photosynthetic apparatus (Nabity et al., 2009, 2013; Bilgin et al., 2010). Recent studies have shown that plant responses to two or more stress factors are complex and not merely the result of concurrent, independent reactions to each kind of stress (Atkinson et al., 2013; Rasmussen et al., 2013; Suzuki et al., 2014; Ramegowda and Senthil-Kumar, 2015). Metabolic and signaling pathways involved in the reaction to complex stresses include specific transcription factors, photosynthetic adjustments, antioxidant protection systems, and biosynthesis of stress hormones (Hirayama and Shinozaki, 2010; Suzuki et al., 2014). The mechanisms accounting for plant reactions to complex stress are still poorly understood, especially at the proteomic level, although understanding this process is critical for many aspects of plant biology.

The chloroplast is a metabolically versatile organelle that besides its photosynthetic functions also plays roles in the biosynthesis of amino acids, hormones, and secondary metabolites (Hossain et al., 2012). In addition, changes in the chloroplast redox status mediate stress responses in the plant cell. Activation of nuclear-encoded stress-related gene expression has been proposed to be regulated by reactive oxygen species (ROS) that originate from chloroplasts (Nomura et al., 2012). Moreover, chloroplast interaction with plasma membrane receptor kinases is one of the key mechanisms that modulate plant stress reactions (Trotta et al., 2014).

Under some kinds of stress, the transcriptional levels of genes for photosynthetic proteins like RuBisCo and RuBisCo activase decrease (Mitra and Baldwin, 2008). Abiotic stresses such as drought interfere with carbon dioxide fixation and ultimately lead to ROS accumulation and damage to chloroplast proteins (Chaves et al., 2009). High-temperature stress causes damage within the electron-transport chain (ETC) of thylakoids and affects RuBisCo activity, resulting in inhibition of photosynthetic activity (Salvucci and Crafts-Brandner, 2004a,b). It is supposed that maintenance of photosynthetic activity is necessary for resistance to abiotic stress factors (Suzuki et al., 2014). However, the mechanisms that induce protein degradation in chloroplasts still lack detailed analysis.

Plant protoplasts are defined as “naked” cells that have their cell wall partially or completely removed and are surrounded only by plasma membrane. They provide an invaluable experimental system for the analysis of protein subcellular localization, protein–protein interactions, and investigation of various biological processes (Zhang et al., 2011; Guo et al., 2012; Pitzschke and Persak, 2012; Burris et al., 2016). Despite the enzymatic treatment, protoplasts are considered to maintain many of the physiological activities of intact plants. However, it was reported that protoplast generation induces oxidative burst (Tiew et al., 2015), activations of hydrolytic enzymes, accumulation of peroxides and phytoalexins (Davey et al., 2005), and induction of genes involved in jasmonic acid (JA) biosynthesis (Xiao L. et al., 2012; Fesenko et al., 2015). A number of stress-mediating AP2/EREBP transcription factors showed differential expression in *P. patens* protoplasts. Moreover, some of them were up-regulated not only under protoplastation, but also under different stresses (Hiss et al., 2014). Nevertheless, little is known about protoplast stress-related response during protoplastation at both proteome and transcriptome level.

*Physcomitrella patens* is a model moss often used for plant systems biology (Rensing et al., 2008; Cove et al., 2009). During the moss life cycle, the haploid generation (the gametophyte) is predominate and goes through two development stages—namely protonemata and gametophores. Protonema filaments serve as a source of protoplasts that are of particular interest as cells from the first hours of regeneration are reprogrammed into protonemal apical stem cells. In this work, we took advantage of this process to study stress responses, as protoplast isolation can be considered to mimic the plasmolysis induced by drought and salinity stress while treatment with Driselase, which digests the cell wall, simulates biotic stress. *P. patens* is of particular interest for studying the effects of stress on the chloroplast proteome due to its high resistance to environmental stresses including drought, salinity, and low temperature (Frank et al., 2005; Minami et al., 2005; Oliver et al., 2005). In a range of proteome studies it has been observed that salinity stress induced up-regulation of light-harvesting chlorophyll a/b-binding proteins, large and small RuBisCo subunits, and a range of other proteins in the chloroplast proteome of moss (Wang et al., 2008). This is consistent with the data for other salt-tolerant organisms (Wang et al., 2013). Under low-temperature stress, the moss down-regulates photosynthetic protein abundance and up-regulates stress-related and some Calvin cycle proteins (Wang et al., 2009).

There is a single study where quantitative proteomic analysis of isolated chloroplasts has been conducted (Mueller et al., 2014) and the proteomes of isolated chloroplasts have been addressed by two studies (Polyakov et al., 2010; Mueller et al., 2014). Here, we performed chloroplast proteome quantification using SWATH-MS to observe changes associated with protoplast isolation. Furthermore, we conducted correlation analysis between quantitative proteomic and RNA-seq transcriptomic data. The abundance of 219 chloroplast proteins changed more than 1.4-fold in protoplasts compared to their levels in protonemal tissue before protoplast isolation. We observed degradation of a significant portion of the chloroplast proteome along with a simultaneous increase in the abundance of some stress-related and ROS detoxifying proteins following the first hour of stress imposed by the protoplast isolation process. There were no genes for which induction at the transcriptomic level preceded an increase in abundance of the corresponding protein. Previously, we found significant changes in the peptidome of moss protoplasts compared with those of protonemata (Fesenko et al., 2015). Increase of the number of peptides of chloroplast proteins is accompanied by suppression of photosynthetic activity. Our findings clearly indicate that the initial chloroplast reaction to combined stress relates to changes at the proteomic level.

## Materials and methods

### Plant material and growth conditions

The protonemata of the moss *P. patens* subsp. *patens* Gransden 2004 were grown on Knop medium with 500 mg/L ammonium tartrate with 1.5% agar (Helicon, Moscow, Russian Federation) in a Versatile Environmental Test Chamber, MLR-352 H (Panasonic, Osaka, Japan) with a photon flux of 61 μmol/m^2^•s during a 16-h photoperiod at 24°C and relative humidity of 50%. For analyses, we used 5-d-old protonema tissue.

### Protoplast preparation

To prepare protoplasts, we used the Liu and Vidali (2011) method with some modifications. Five-day-old protonema filaments were harvested with a spatula from the agar surface, and 1 g well-drained protonema tissue was placed in 14 mL 0.5% (w/v) Driselase (Sigma-Aldrich, St. Louis, MO, USA) in 0.48 M mannitol and incubated for 60 min with constant shaking in darkness. Then, the suspension was filtered through 100-μm steel mesh (Sigma-Aldrich), and the protoplasts obtained were then precipitated by centrifugation in 50-mL plastic tubes using a swinging bucket rotor at 100 × *g* for 5 min. Protoplasts were washed twice with 0.48 M mannitol with centrifugation under the same conditions and sedimented again. The supernatant was removed, and the protoplast pellet was used for chloroplast preparation.

### Isolation of chloroplasts from moss protonemata and protoplasts

The 5-d-old protonema tissue was placed in chilled buffer A (50 mM HEPES-KOH, pH 7.5, 330 mM sorbitol, 2 mM EDTA, and 0.4 mM phenylmethylsulfonyl fluoride) and ground with an immersion homogenizer at 4°C. Then, the suspension was filtered through double-folded Miracloth (Calbiochem). This filtration and homogenization was repeated twice. The filtrate was then centrifuged at 1200 × *g* for 3 min in 50-mL plastic tubes using a bucket rotor. The pellet was resuspended in a small volume of buffer A and fractionated by centrifugation in a bucket rotor at 3800 × *g* for 10 min in a 10%–40%–85% stepwise Percoll (Sigma-Aldrich) gradient in 15-mL plastic tubes. Intact chloroplasts between the 40% and 85% Percoll layers were gathered, washed with buffer A, and centrifuged at 1200 × *g* for 3 min in 15-mL plastic tubes (Falcon) in a bucket rotor. The resulting chloroplast pellet was used for protein extraction.

Alternatively, protoplasts prepared as described in the previous section were resuspended in buffer A (50 mM HEPES-KOH, pH 7.5, 330 mM sorbitol, 2 mM EDTA, and 0.4 mM phenylmethylsulfonyl fluoride) and filtered through a double layer of Miracloth (Calbiochem Behring, La Jolla, CA, USA). Protoplast disintegration was confirmed with a light microscope. The subsequent chloroplast isolation was performed as described above, beginning with fractionation on the Percoll gradient.

### Extraction of chloroplast proteins

Proteins were extracted using a phenol extraction procedure. Three volumes of ice-cold extraction buffer (500 mM Tris-HCl, pH 8.0, 50 mM EDTA, 700 mM sucrose, 100 mM KCl, 1 mM PMSF, 2% 2-mercaptoethanol, 1% Triton X-100) were added to the chloroplast pellet followed by incubation for 10 min on ice. Then, an equal volume of ice-cold Tris-HCl (pH 8.0)-saturated phenol was added, and the mixture was vortexed and incubated for 10 min with shaking. After centrifugation (10 min, 5500 *g*, 4°C), the phenol phase was collected and re-extracted two times with extraction buffer. Proteins were precipitated from the final phenol phase with 3 volumes of ice-cold 0.1 M ammonium acetate in methanol overnight at −20°C and centrifuged for 10 min at 5500 *g* and 4°C. The pellets were rinsed with ice-cold 0.1 M ammonium acetate in methanol three times and with ice-cold acetone containing 13 mM DTT once and then dried. Pellets were solubilized in sample buffer (8 M urea, 2 M thiourea, 10 mM Tris-HCl, pH 8.0). Protein content was estimated using Bradford method.

### In-solution trypsin digestion of chloroplast protein

An equal amount of protein from each sample was taken to be analyzed. Ammonium bicarbonate was added to each sample to a final concentration of 25 mM. To reduce disulphide bonds, DTT was added to each sample to 5 mM, and the samples were incubated for 30 min at 56°C, then cooled to room temperature and alkylated with 10 mM ioadacetamide for 20 min in darkness. The alkylated samples were diluted 6 times with 25 mM ammonium bicarbonate. Trypsin (Promega, USA) was added (0.01 μg per 1 μg protein), and the samples were incubated for 12 h at 37°C. The reaction was stopped by 5% TFA. The tryptic peptides derived from protonemal and protoplast proteins were desalted on microcolumns C-18 (Supelco, USA), vacuum-dried, and stored at −70°C.

### LC-MS analysis and protein identification

Analysis was performed on a TripleTOF 5600+ mass-spectrometer with a NanoSpray III ion source (ABSciex, Canada) coupled to a NanoLC Ultra 2D+ nano-HPLC system (Eksigent). The HPLC system was configured in trap-elute mode. Sample loading buffer and buffer A was 98.9% water, 1% methanol, and 0.1% formic acid (v/v). Buffer B was 99.9% acetonitrile and 0.1% formic acid (v/v). Samples were loaded on a trap column Chrom XP C18 3 mm 120 Å 350 mm^*^0.5 mm (Eksigent, Dublin, CA) at a flow rate of 3.5 μL/min over 10 min and eluted through the separation column 3C18-CL-120 (3 μm 120 Å) 0.075 mm^*^150 mm (Eksigent, Dublin, CA) at a flow rate of 300 nL/min. The gradient was from 5 to 40% buffer B in 120 min. The column and the precolumn were regenerated between runs by washing with 95% buffer B for 7 min and equilibrated with 5% buffer B for 25 min. Between the samples, to ensure the absence of carryover, both the column and the precolumn were thoroughly washed with a blank injection trap-elute gradient that included 5–7-min 5-95-95-5% B waves followed by 25 min 5% B equilibration.

Mass spectra were acquired in a positive ion mode. The information-dependent mass-spectrometry experiment included 1 survey MS1 scan followed by 50 dependent MS2 scans. MS1 acquisition parameters were mass range for analysis, and subsequent ion selection for MS2 analysis was 300–1250 m/z; signal accumulation time was 250 ms. Ions for MS2 analysis were selected on the basis of intensity with the threshold of 400 cps and the charge state from 2 to 5. MS2 acquisition parameters were as follows: resolution of quadrupole was set to UNIT (0.7 Da), measurement mass range was 200–1800 m/z, optimization of ion beam focus was to obtain maximal sensitivity, signal accumulation time was 50 ms for each parent ion. Collision-activated dissociation was performed with nitrogen gas with collision energy ramping from 25 to 55 V within 50 ms signal accumulation time. Analyzed parent ions were sent to dynamic exclusion list for 15 s to obtain the next MS2 spectra of the same compound around its chromatographic peak apex (minimum peak width throughout the gradient was about 30 s).

For protein identification, data files were analyzed with ProteinPilot 4.5 revision 1656 (ABSciex, Canada) using search algorithm Paragon 4.5.0.0 revision 1654 (ABSciex, Canada) and a standard set of identification settings. The database was obtained by combining UniProtKB (organism *P. patens*) and the protein database from cosmoss.org V1.6 (Zimmer et al., 2013). In the case of 90% sequence identity, only the entries from the cosmoss.org database were taken. The following parameters were used: alkylation of cysteine—iodoacetamide, trypsin digestion, TripleTOF 5600 equipment, species: none, thorough search with additional statistical FDR analysis. Peptide identifications were processed with default settings using the ProteinPilot software built-in ProGroup algorithm. The final protein identification list was obtained with the threshold reliable protein ID unused score calculated by ProteomicS Performance Evaluation Pipeline Software (PSPEP) algorithm for 1% global FDR from fit.

### Quantitative LC-MS analysis

Quantitative LC-MS protein analysis was performed on the basis of DIA SWATH technology (Gillet et al., 2012). Three biological and two technical replicates were performed for protonemal and protoplast samples. Raw data were obtained by triplicate injection of each sample with LC parameters identical to those of IDA experiments. HPLC configuration was the same as described for protein identification. The SWATH acquisition parameters were as follows: one 50 ms MS1 scan for m/z 100–2000, followed by 32 SWATH windows 20 a.m.u width covering parent ion mass range from 400 to 1000 a.m.u. MS2 SWATH scans were collected in high sensitivity mode for mass range 200–1800 and 100 ms single window accumulation time. The total cycle time was ~3.3 s.

To process SWATH data, protein identification lists (.group files) obtained for IDA experiments were translated into an ion library with PeakView 2.0 (ABSciex, Canada) SWATH processing tools for the number of proteins according to 1% FDR from fit for the corresponding .group file and reported peptide confidence equal to 99 with the exclusion of shared and modified peptides (except for carbamylation of cysteines). The ion library was used to obtain extracted ion chromatograms for the corresponding transitions from SWATH data files with the following parameters: 1000 peptides per protein and 1000 (all observed) fragments per peptide, extraction window 15 min, mass window 50 ppm. The next step suggested by the manufacturer (ABSciex, Canada) is direct comparison of summary peptide intensities (as summary fragment intensities) per protein; however, this method almost completely lacks quality control. To enhance reliability, we filtered extracted ion chromatogram data starting from the ion level (that is for each pair of parent and fragment ions in the ion library separately) for reliably quantifiable proteins using a homemade script in R. The algorithm included scaling normalization and averaging of three technical repeats per sample [each biological sample was treated in duplicate (triplicate for protonema) and each obtained tryptic peptide sample was analyzed in triplicate, thus this step allows for injection and LC-MS signal reproducibility], exclusion of peptides with <3 quantified fragments, and exclusion of proteins with <3 quantified peptides, followed by separation of quantifiable transitions. The latter was based on the assumption of the proportionality of fragment ion intensity to parent ion intensity and, consequently, the proportionality of fragment intensity changes between different samples (Toprak et al., 2014). Quantifiable fragment ions were selected on the basis of trend searches within all fragments on the basis of normalized spectral contrast angle analysis with iterative searches of fragment clusters. The minimal number of fragments that must follow the same trend between samples to be used as “reliable” was set at 3.

After fragment filtering, all proteins with fewer than 3 peptides were excluded. This was followed by renormalization of different LC-MS repeats of the same sample and averaging, and then by the second normalization and averaging step between technical repeats of trypsinolysis of the same sample and a third normalization and averaging step within sample type. To obtain final results, biological samples were cross scale-normalized on the basis of the assumption that most of the cell proteins in any pair of samples were independent (that is, the scaling should set the minimum average difference between proteins for a pair of samples).

To calculate protein fold-change results, logarithm with the base *B* (was chosen to obtain the best scaling) was taken for all fragment intensities. The logarithm results for each fragment were averaged within a sample (LC-MS repeats^*^trypsinolysis repeats^*^technical repeats) to obtain peptide logarithm results. Each protein result was calculated as a median of the top 3 most intense (based on total intensity of all fragmentation spectra for each peptide) of its tryptic peptides. Fold change for a protein was calculated as the difference in median values between samples raised to power *B*.

For proteins that could not be quantified by SWATH we used the quantitative assessment method emPAI (Exponentially Modified Protein Abundance Index; Ishihama et al., 2005). Scaffold v 4.2.1 (Proteome Software, USA) was used to calculate the emPAI algorithm.

### Proteomic analysis with MRM-MS

Quantitative LC-MS protein analysis was performed on the basis of a scheduled MRM methodology on QTRAP 4500 (Sciex, USA) triple quadrupole mass spectrometer equipped with a NanoSpray III ion source (Sciex, USA) coupled to an expert NanoLC 400 nano-HPLC system (Eksigent, USA). To obtain transition list for proteins of interest, peak lists with protein identification (.group files) obtained for IDA experiments were loaded into Skyline software, where no more than 10 unique peptides per protein with global FDR rate from fit according to PSPEP were selected with additional constrains on peptide length (5–30 amino acid residues), cleavage (fully tryptic peptides without missed cleavage sites or potential ragged ends) and modifications (only carbamidomethylation of cysteines allowed). For each precursor of charge 2, 3, or 4, 3, or 4 most intense fragment ions were selected. Then, the obtained preliminary list of peptides was divided into several scheduled MRM transition lists on the basis of retention time and scan time of each individual peptides was no <20 ms. This preliminary lists were analyzed in the same HPLC setup, as for protein identification, except for the length of chromatographic gradient which was 30 min. Mass-spectra were acquired in positive ion mode in scheduled MRM method with 2.4 s target scan and 420 s MRM detection window (Q1 and Q3 resolution ≪unit≫ (0.7 Da FWHH), pause between mass ranges 2 ms). Obtained preliminary MRM transition list was validated manually in Skyline software, i.e., peptides with low signal intensity or missing chromatographic peak were excluded. Finally, no more than 3 best-flyer peptides per target protein were retained. Raw data was obtained by duplicate injection of each sample for one final list of transitions with the same chromatography and mass-spectrometry setup except 240 s MRM detection window.

Processing of the data included peak selection (with manual review for interference and missing signals), peak integration and export utilizing Skyline software. Further analysis was performed by a homemade script in R. The algorithm included scaling normalization of LC-MS replicates on peptide level and of each set of samples on protein level (based on 2 most intense peptides). Differences were considered to be statistically significant if FDR (obtained by Benjamini–Hochberg correction procedure for multiple comparisons) after Student's *t*-test across 3 biological replicates was below 0.05.

### Global gene expression and differential gene expression analysis

In the previous research, we performed RNA-seq analysis of the three cell types of moss protonemata, gametophores, and protoplasts (Fesenko et al., 2015). To evaluate the gene expression level in FPKM (Fragments Per Kilobase Of Exon Per Million Fragments Mapped), the produced.bam file was processed with the Cufflinks utility (Trapnell et al., 2010), and we used HTSeq to count the number of mapped reads for each gene. For analysis of differential expression, the edgeR (Robinson et al., 2010) package was used and the analysis was performed according to the recommendations in the edgeR vignette. We used read count per gene data as input for edgeR. For the subsequent analysis, differential expression was considered significant if *p*-value was greater than the false discovery rate (FDR) after Benjamini–Hochberg correction for multiple-testing and FDR < 0.05. In addition, a more stringent rule of |log2(FPKMy/FPKMx)| > 2 was used in order to differentiate genes with high levels of differential expression.

### Validation of RNA-Seq data by real time RT-PCR

Total RNA was isolated from protonema tissues and protoplast cells as previously described (Cove et al., 2009). The quality and quantity of the extracted total RNA was initially evaluated by electrophoresis in agarose gels with ethidium bromide staining. Quantification of the total RNA in the sample was carried out with the Quant-iT RNA Assay Kit, 5–100 ng kit in a Qubit fluorometer (Invitrogen, USA). For qPCR, cDNA was synthesized by the MMLV-RT kit (Evrogen, Moscow, Russia) according to the manufacturer's instructions. PCR experiments were carried out using three biological and three technical replicates. For each of the three technical repeats, cDNA corresponding to 2 μg of total RNA was used. Melting curve analysis was performed for each primer pair before further analyses. Real-time PCR was performed using the qPCRmix-HS SYBR system and the LightCycler® 96 Real-Time PCR Detection System (Roche, Mannheim, Germany). The *AdePRT* was used as a reference gene (Le Bail et al., 2013). Relative fold differences for each sample were calculated using the ΔΔCt method (Thimm et al., 2004). The primers for subsequent qPCR reactions are listed in Supplementary Table 1.

### Analysis of photosynthetic activity of *P. patens* protonemata and protoplasts

The measurements were carried out as previously described (Fesenko et al., 2015). The rate of electron transport was estimated on the basis of data for light dependence of quantum efficiency of PSII (Genty et al., 1989). Protonema tissue was exposed to actinic light of each intensity for 1 min starting from the lower intensity.

### Availability of supporting data

The mass spectrometry proteomics data have been deposited to the ProteomeXchange Consortium (Vizcaino et al., 2014) via the PRIDE partner repository with the dataset identifier PXD002866 and 10.6019/PXD002866. The MRM data sets have been submitted via the PRIDE partner repository with the dataset identifier PXD005017.

## Results

### Overview of comparative quantitative proteomic analysis in moss chloroplasts

**Figure 1:**
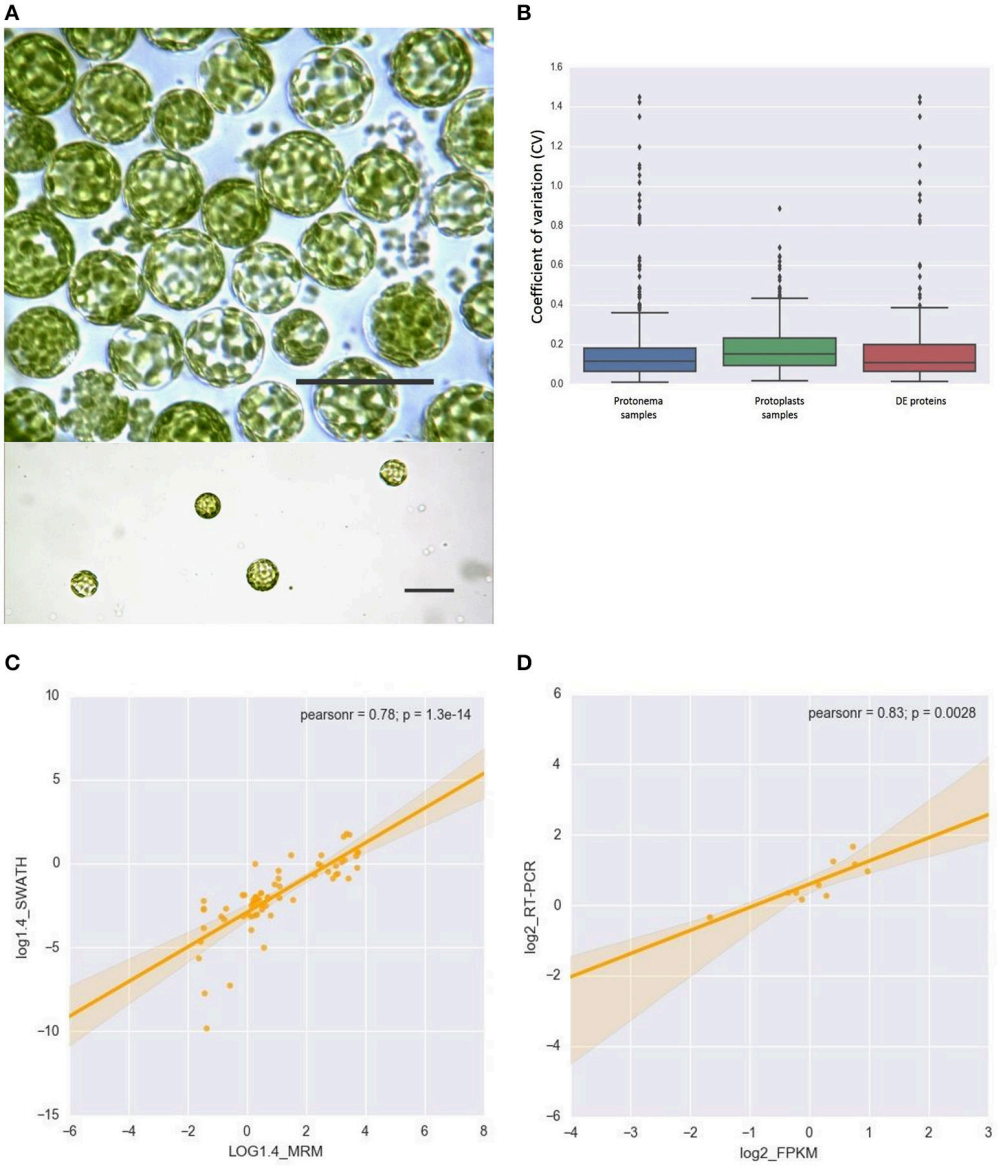
**(A)** Light micrographs of protoplasts used in this study. Scale bars correspond to 50 μm. **(B)** Box plot and 5–95 percentile whiskers illustrating the distribution of the coefficient of variation (CV) values of 503 SWATH-MS quantified proteins: protonema samples, protoplast samples, Differentially Accumulated proteins. The majority of proteins had a time course CV < 0.3. **(C)** The correlation analysis of the chloroplast proteins quantified by SWATH-MS and MRM-MS. The x-axis displays the protein fold changes quantified by MRM and the y-axis displays the protein fold changes quantified by SWATH. **(D)** Correlation of the fold change analyzed by RNA-Seq (x-axis) with the data obtained using real time PCR (y-axis).

We used protoplasts of the moss *P. patens* to track changes in chloroplast proteome under stress conditions (Figure 1A). Protoplasts were isolated from protonemal tissue using Driselase, a natural enzyme mixture containing laminarinase, xylanase and cellulase activities (Noguchi et al., 1978). This process imposes stress on plant cells, due to both loss of the cell wall and the effects of Driselase itself. We isolated chloroplasts from moss protonematal tissue and from protoplasts, to analyze the chloroplast proteome before and after stress, respectively. Using the SWATH-MS approach, we quantified 503 proteins, 479 of which were annotated as chloroplast proteins and 24 were classified as mitochondrial, cytosolic, and membrane proteins (Supplementary Table 2, lists 1, 2, and 3). The variation analysis of proteins, quantified by SWATH, and shared across all biological repeats, was based on the coefficient of variation (CV). The median CVs were 0.112 and 0.150 for protonema and protoplast chloroplast proteomes, respectively (Figure 1B).

To validate SWATH-MS data, we used multiple-reaction monitoring mass spectrometry analysis (MRM-MS), which allows sensitive, precise quantitative analyses of peptides, and proteins from which they are derived (Aebersold et al., 2013; Doerr, 2013). The MRM data structure is similar to that of SWATH-MS and the qualitative and quantitative information extracted from SWATH-MS are ideal to verify by MRM-MS assays (Hou et al., 2015). When the protein abundance ratios of 65 SWATH-MS quantified proteins (adequately representing the major groups of chloroplast proteins) were plotted against that those derived from MRM-MS, the Pearson correlation coefficient was 0.78 (Figure 1C; Supplementary Table 3). This result indicates that protein abundances estimated by the different approaches have a good correlation.

For the proteins identified but not quantified by SWATH, we used the emPAI method (Ishihama et al., 2005) to evaluate their abundance. Using both these methods, we quantified 1932 plastid proteins (Supplementary Tables 2, 4). We found that 577 proteins identified in our study have been previously reported in a quantitative proteomic study of moss chloroplasts (Mueller et al., 2014). Thus, ~80% chloroplast proteins (577 from 687 proteins) from the previous study have been identified in our dataset (Supplementary Figure 1; Supplementary Table 5). The difference in gene number between our and the previously published (Mueller et al., 2014) datasets can be explained by differences in sample preparation (Leon et al., 2013) and the types of mass-spectrometry devices that were used.

**Figure 2:**
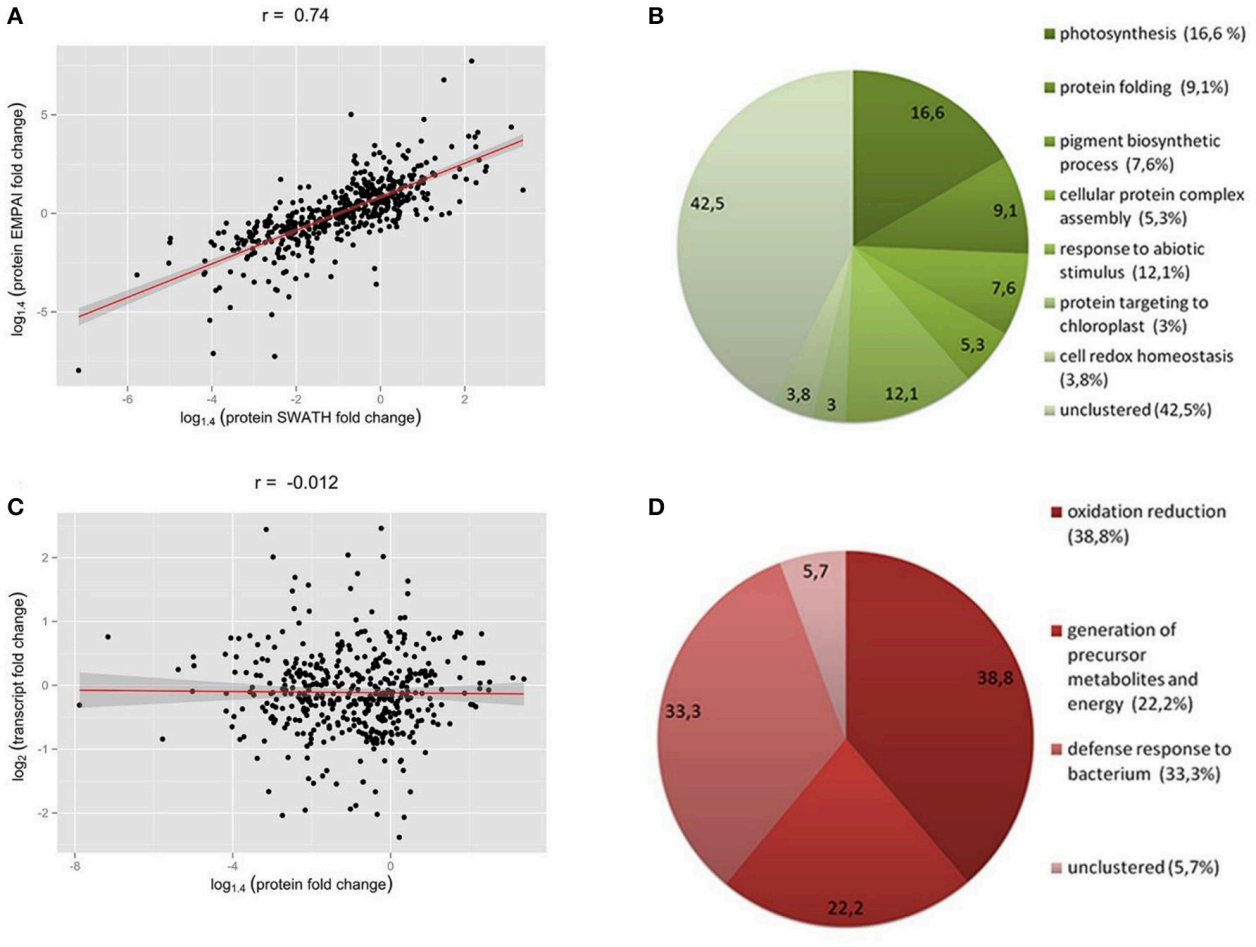
**(A)** Correlation between SWATH- and emPAI-derived protein fold change **(A)**, r, Pearson correlation coefficient. **(B,D)** Functional annotation of differentially accumulated proteins (DAPs) in chloroplasts of protoplasts compared to protonemal tissue. GO biological process terms for down-regulated proteins in protoplasts **(B)** and up-regulated proteins **(D)**. Figures in brackets and on the diagram indicate % of all clusterized genes by topGO (132 down-regulated proteins and 18 up-regulated proteins). **(C)** SWATH-derived protein fold change and transcript FPKM-derived fold change for corresponding genes.

We found a high correlation between the SWATH-MS and emPAI data (Figure 2A). However, we used the emPAI data only to investigate metabolic pathway changes in chloroplasts because of the advantages of the SWATH-MS approach in protein quantification (Gillet et al., 2012). To annotate the predicted moss proteins, we used BLAST homology searches against the *Arabidopsis thaliana* TAIR database.

We found that 219 proteins were up- or down-regulated in protoplasts relative to protonema (>1.4-fold change; *p* < 0.05) and designated them as differentially accumulated proteins (DAPs; Table 1; Supplementary Table 2, list 4). According to our data, the abundance of 23 DAPs increased and that of 196 decreased. For functional annotation of DAPs, we used the topGO tool (Figures 2B,D).

**Table 1:** **List of top 15 up- and down-regulated proteins in protoplasts**.

**Gene ID**	**Description**	**Homolog gene name**	**log_1.4_FC**
**UP REGULATED**			
Pp1s69_154V6	Acyl carrier protein	ACP4	3.101
Pp1s39_428V6	Malate glyoxysomal precursor	PMDH2	2.487
Pp1s159_143V6	Chloroplast protein CP12	CP12, CP12-1	2.339
Pp1s38_300V6	Malate glyoxysomal precursor	PMDH2	2.261
PhpapaCp013	ATP-dependent protease proteolytic subunit	CLPP1, PCLPP	2.228
Pp1s214_32V6	Adenylate kinase	AMK2	2.161
Pp1s199_33V6	Isocitrate dehydrogenase (nad+)	IDH-V	2.092
Pp1s307_65V6	Porphobilinogen deaminase	HEMC, RUG1	2.035
Pp1s137_140V6	5-enolpyruvylshikimate-3-phosphate synthase		1.877
Pp1s30_345V6	2-cys peroxiredoxin-like protein		1.877
Pp1s12_401V6	Dehydroascorbate reductase	DHAR3	1.778
Pp1s257_45V6	2-cys peroxiredoxin bas1	2-Cys Prx B, 2CPB	1.757
Pp1s8_193V6	Thioredoxin x	ATHX, ATHP	1.690
Pp1s132_175V6	Phosphoribulokinase precursor	PRK	1.631
**DOWN REGULATED**			
PhpapaCp040	PSI P700 apoprotein A2	PSAB	−10.6813
PhpapaCp039	PSI P700 apoprotein A1	PSAA	−9.8149
Pp1s163_57V6	Glutamate malate translocator	DCT, DIT2.1	−7.88186
PhpapaCp043	PSII 43 kDa protein	PSBC	−7.74407
PhpapaCp044	PSII D2-protein	PSBD	−7.27874
Pp1s66_172V6	Glutathione s-transferase	ATGSTF10, ATGSTF4, ERD13, GSTF10	−7.16067
Pp1s270_50V6	Plastidic atp adp transporter	ATNTT1, NTT1	−5.77673
PhpapaCp046	PSII D1-protein	PSBA	−5.64896
PhpapaCp012	PS II P680 chlorophyll A apoprotein	PSBB	−5.43628
Pp1s145_122V6	MKP11.2; expressed protein [*Arabidopsis thaliana*]	ENH1	−4.99762
Pp1s41_264V6	PS II 10 kda polypeptide	PSBR	−4.98293
PhpapaCp081	NADH dehydrogenase 49 kDa subunit	NDHH	−4.63947
Pp1s249_62V6	Peptidyl-prolyl cis-trans isomerase	ATCYP1, ROC5	−4.16531
Pp1s28_391V6	K2A11.7; expressed protein [*Arabidopsis thaliana*]		−4.16214

The most highly represented GO terms for down-regulated proteins were photosynthesis (16.6 %, GO:0015979), response to abiotic stimulus (12.1%, GO:0009628), protein folding (9.1%, GO:0006457), pigment biosynthetic process (7.6%, GO:0046148), cellular protein complex assembly (5.3%, GO:0043623), cell redox homeostasis (3.8%, GO:0045454), and protein targeting to chloroplast (3%, GO:0045036). For up-regulated proteins, the most represented GO terms were oxidation reduction (38.8%, GO:0055114), defense response to bacterium (33.3%, GO:0042742), and generation of precursor metabolites and energy (22.2%, GO:0006091).

Previously, we performed analysis of transcriptomes for three types of moss cells—protonema, gametophores, and protoplasts (Fesenko et al., 2015). Using these data, we estimated the transcriptional level of 428 nuclear-encoded chloroplast proteins that had been quantified with the SWATH-MS approach.

### Quantification of proteins of the photosynthetic apparatus

**Figure 3:**
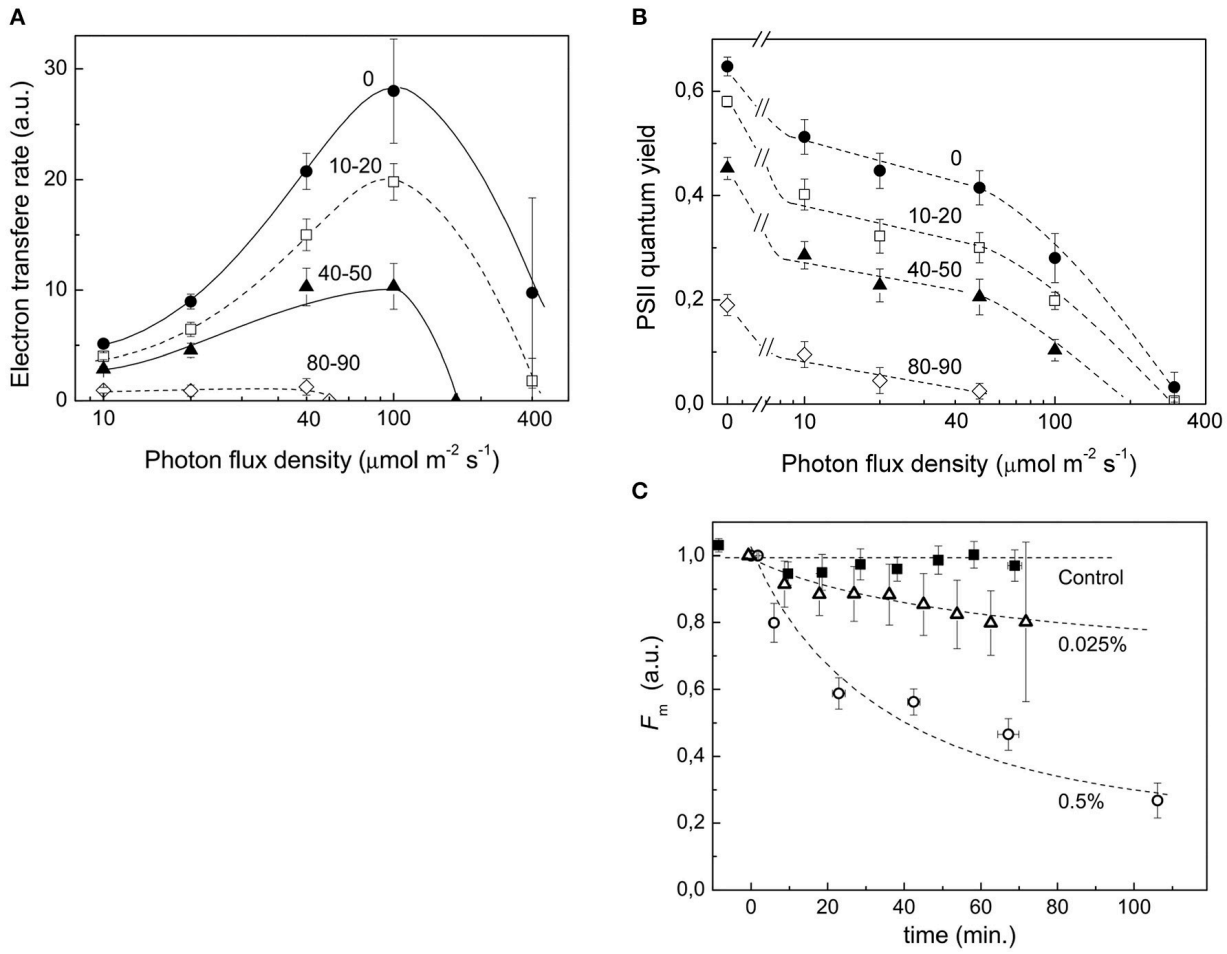
**Decrease in functional activity of chloroplasts during Driselase treatment. (A)** Linear electron transport rates were calculated from PSII effective quantum yields at different actinic light intensities. The numbers near the curves represent the time (in minutes) of exposure of protonema to 0.5% (w/v) Driselase. The average values ± standard errors from 4 to 7 samples are presented. **(B)** Decrease in PSII effective quantum yields at different actinic light intensities. The numbers near the curves represent the time (in minutes) of exposure of protonema to 0.5% (w/v) Driselase. The average values ± standard errors from 4 to 7 samples are presented. **(C)** Fm values over time of *P. patens* protonema exposed to Driselase. The numbers near curves indicate concentration of Driselase (w/v), “control” indicated protonema incubated in 0.48 M mannitol. The average values ± standard errors from 6 to 14 samples are presented.

Stress conditions inhibit the photosynthetic activity of a plant cell (Nouri et al., 2015). During protonema maceration by Driselase, we observed inhibition of chloroplast functional activity (Figures 3A,B). The quantum yield of photochemical reaction in PSII declined for dark and light acclimated protonema (Figure 3C), leading to slowing of the electron outflow from the ETC (Figure 3A). Total chlorophyll fluorescence also declined during maceration (Figure 3C).

These processes reflect simultaneous decreases in abundance of chlorophyll-containing ETC components like PSII proteins and antenna light-harvesting complexes (LHC) in protoplasts. Accordingly, there was significant down-regulation of proteins representative for photosystems I and II (Supplementary Table 6) according to SWATH-MS experimental data. We observed decreases in abundance of PSI and PSII reactive center proteins including psaA, psaB, psaC, and psaE (Pp1s334_17V6, Pp1s319_36V6) and psbA, psbB, psbC, psbD, and psbE, respectively, as well as of the oxygen-evolving complex PsbO2 (Pp1s60_65V6, Pp1s421_3V6, Pp1s306_84V6), Psb P-1 (Pp1s63_71V6, Pp1s135_79V6, Pp1s75_141V6), Psb R (Pp1s41_264V6), and PsbP domain-containing protein 1 (Pp1s88_182V6). The abundance of proteins of the LHC of PSI (Lhca5, Pp1s284_6V6) and PSII (Lhcb2.1, Pp1s76_196V6, Pp1s13_200V6, Pp1s52_157V6, Pp1s27_97V6; Lhcb2.2, Pp1s252_28V6; Lhcb3, Pp1s254_3V6, Lhcb5, Pp1s628_7V6) decreased as well. In addition, we quantified five subunits of the NAD(P)H-quinone oxidoreductase complex, which carries out a part of PSI cyclic electron transport process. The abundance of H (ndhH), I (ndhI), J (ndhJ), K (ndhK), M (Pp1s230_42V6), N (Pp1s230_42V6), S (Pp1s123_43V6) subunits decreased. The same was true for the proteins homologous to cyclophilin 38, which is essential for the assembly and maintenance of PSII supercomplexes (Pp1s211_59V6 and Pp1s90_50V6; Fu et al., 2007).

Other proteins of the chloroplast ETC, such as apocytochrome f (petA), cytochrome b6-f complex iron-sulfur subunit (Pp1s35_78V*6*, petC), Rieske (2Fe-2S) domain-containing protein (Pp1s270_57V6), and plastocyanin (petE—Pp1s254_25V6, Pp1s3_520V6, Pp1s27_130V6) were also a subject to degradation. We analyzed the changes in protein abundance of ATP synthase, a complex localized in thylakoid membranes. In chloroplasts, there was an ATP synthase complex of F type. We observed a decrease in the stromal F_1_ component of ATP synthase, catalytic atpA subunit (Alpha-subunit), and atpB subunit (Beta-subunit). The gamma subunit (Pp1s372_16V6, Pp1s287_61V6, Pp1s35_234V6), involved in forming the “stalk” connecting the stromal part of the ATP synthase complex and membrane anchored CF_0_, also experienced degradation. AtpF is a part of the F_0_ component and also decreased in protoplasts over time.

According to our data, the abundance of proteins in the Calvin cycle did not change except for that of phosphoribulokinase (Pp1s132_175*V6*). We also found up-regulation of the small protein A9RS50 (Pp1s159_143V6), homologous to Arabidopsis protein CP12-1 (*AT2G47400*), a vital component for building a complex between glyceraldehyde-3-phosphate dehydrogenase (GAPDH) and phosphoribulokinase (PRK; Marri et al., 2010).

### Analysis of abundance of chloroplast protease

We showed that a substantial portion of the chloroplast proteome degraded in protoplasts. Accordingly, we analyzed the abundance change of the main proteases involved in degradation or processing of proteins inside chloroplasts (Supplementary Table 6). We observed a decrease in abundance of Pp1s197_46V6 protein, a putative carboxyl-terminal-processing peptidase 1, CTPA1, involved in processing the D1 subunit of the PSII reaction center (Che et al., 2013). We also noticed down-regulation at the protein level of the proteases involved in the PSII repair cycle: Pp1s118_166V6 and Pp1s156_30V6 (FTSH8), Pp1s44_78V6 (FTSH5, VAR1), and Pp1s160_79V6 (putative Deg P1; Kapri-Pardes et al., 2007; van Wijk, 2015). By contrast, the abundance of ATP-dependent Clp protease (clpP) increased.

### Quantification of stress-related proteins

ROS such as superoxide, peroxide, and singlet oxygen accumulate within the cell as a result of biotic and abiotic stress (Choudhury et al., 2013; You and Chan, 2015). Accordingly, plants have evolved antioxidant defense mechanisms to protect themselves from partial or severe oxidation of cellular components. Among the DAPs we identified were those that detoxify ROS. One of the key mechanisms protecting chloroplasts from ROS is the ascorbate-glutathione cycle (Foyer and Noctor, 2011). Ascorbate peroxidase is an important part of this cycle due to its ability to reduce H_2_O_2_ to water with concomitant generation of dehydroascorbate (Asada, 1999). We observed decreased abundance of Pp1s424_33V6 protein, homologous to thylakoid ascorbate peroxidase of Arabidopsis (AT1G77490) in protoplasts. However, a protein with putative dehydroascorbatereductase (DHAR) activity, Pp1s12_401V6 (putative glutathione-dependent dehydroascorbate reductase 3), was up-regulated in protoplasts.

We also quantified seven proteins from another class of antioxidant proteins—the peroxiredoxin family. For Pp1s95_45V6, Pp1s233_104V6, and Pp1s30_253V6 proteins, which belong to the PrxQ class of atypical peroxiredoxins, and for Pp1s1_230V6 and Pp1s52_248V6 proteins, which are homologous to peroxiredoxin-like protein, we did not detect any significant change in abundance. By contrast, the abundance of Pp1s30_345V6 and Pp1s257_45V6, from a typical peroxiredoxin class (2-Cys peroxiredoxins), increased in protoplasts. Proteins that influence the redox status of peroxiredoxins were also more abundant, for example, thioredoxin protein Pp1s8_193V6, an electron donor for peroxiredoxins.

In the photosystem reaction centers and antenna proteins, carotenoids (β-carotene and lutein) prevent singlet oxygen production via quenching the chlorophyll triplet state (Ramel et al., 2012). Besides this constitutive protective mechanism there is also a light-regulated and reversible reaction to neutralize singlet oxygen that occurs in the PSII antenna via the violaxanthin cycle (Vass, 2012). We detected down-regulation of proteins that are part of that cycle including zeaxanthin epoxidase (Pp1s91_16V6) and violaxanthin de-epoxidase (Pp1s161_120V6) in protoplasts.

Proteins of light- and dark-dependent phases of photosynthesis are also part of the stress reaction (Kangasjarvi et al., 2012). We observed an increase in abundance of phosphoribulokinase involved in the defense reactions (defense response to bacterium GO:0042742/response to bacterium GO:0009617; response to temperature stimulus GO:0009266; response to abiotic stimulus GO:0009628) in protoplasts.

Besides the proteins mentioned above, we detected increases in abundance of such proteins as Pp1s372_19V6 (a possible Co-chaperone GrpE family protein), Pp1s307_65V6 (hydroxymethylbilane synthase), and a range of other stress-related proteins (Supplementary Table 2).

### Changes of protein abundance in metabolic pathways of chloroplasts

We next used the SWATH-MS and emPAI quantification data to assess the effect of stress conditions on the main chloroplast metabolic pathways. The correlation index between the SWATH-MS and emPAI data was rather high (*r* = 0.74), so we combined these data to improve accuracy and better understand the underlying processes (Figure 2A).

Based on the proteome quantification data, we concluded that several metabolic pathways were down-regulated in chloroplasts isolated from protoplasts (Supplementary Table 7). For example, the abundance of enzymes involved in the biosynthesis of tocopherols, carotenoids, and chlorophyll decreased. For instance, the abundance of Pp1s196_120V6 protein (a putative phytoene synthase, EC:2.5.1.32), which uses geranylgeranyl pyrophosphate as a substrate for further reactions in the carotenoid biosynthesis pathway was down-regulated according to emPAI. We quantified eight other proteins involved in the biosynthesis of β- and γ-carotenoids (Supplementary Table 7; List 1—CARATENOID BIOSYNTHESIS) and five of them were significantly down-regulated.

Plastidial isoprenoids, such as isoprene, monoterpenes, diterpenes, plastoquinone, phylloquinone, carotenoids, chlorophylls, and tocopherols are synthesized via plastidic 2C-methyl-D-erythritol 4-phosphate pathway (MEP; Hemmerlin et al., 2012; Vranova et al., 2013). We detected a decrease in the abundance of 1-deoxy-D-xylulose-5-phosphate synthase (DXS), which is responsible for the first step in plastidic isoprenoid synthesis (Banerjee and Sharkey, 2014; Supplementary Table 7. List 2. TERPENOID BACKBONE BIOSYNTHESIS; List 2. TERPENOID QUINONE BIOSYNTHESIS). The correlation between the expression level of DXS and the levels of several plastidic isoprenoids was found in transgenic Arabidopsis plants with over- or under- expression of this enzyme (Estevez et al., 2001). Also, Pp1s100_107V6 (geranylgeranyl reductase, EC:1.3.1.83) that provides phytol for both tocopherol and chlorophyll synthesis (Tanaka et al., 1999) and Pp1s169_113V6 (2-phytyl-1,4-beta-naphthoquinone methyltransferase EC:2.1.1.163 2.1.1.201) responsible for biosynthesis of phylloquinone, the acceptor of PSI electrons, decreased in abundance (Supplementary Table 7. List 2. TERPENOID BACKBONE BIOSYNTHESIS; List 3. TERPENOID QUINONE BIOSYNTHESIS). At the same time, we observed that some proteins in terpenoid biosynthesis pathway were up-regulated (Supplementary Table 3). The most up-regulated proteins were 2-C-methyl-D-erythritol 2,4-cyclodiphosphate synthase (MEcDP synthase, EC:4.6.1.12) and isopentenyl-diphosphate delta-isomerase (IPPI; EC:5.3.3.2) (Supplementary Table 7). The MEcDP synthase catalyzes the conversion of 4-diphosphocytidyl-2-C-methyl-D-erythritol 2-phosphate (CDP-ME2P) to 2-C-methyl-D-erythritol 2,4-cyclodiphosphate (MEcDP; Vranova et al., 2013). It has been shown that MEcDP induces the expression of a nuclear stress-related gene that encodes a chloroplast-localized protein in the oxylipin pathway (Xiao Y. et al., 2012). MEcDP accumulation also activates salicylic acid—induced defense responses in *Arabidopsis thaliana* and enhanced resistance to the phloem-feeding aphid *Brevicoryne brassicae* (Gonzalez-Cabanelas et al., 2015). We also observed that Pp1s28_399V6 (IPPI) is up-regulated in protoplasts (Supplementary Table 7). Previously, Nakamura et al. showed that transcription of IPPI plastid isoform is induced in response to abiotic stress (Nakamura et al., 2001).

In addition, we detected quantitative changes in some proteins involved in the biosynthesis of porphyrins and chlorophylls. The abundance of protoporphyrinogen oxidase (E.C. 1.3.3.4; Pp1s28_304V6, Pp1s45_76V6), the last common enzyme in the biosynthesis pathways of heme and chlorophyll (Che et al., 2000), decreased (Supplementary Table 7. List 4. PORPHYRIN AND CHLOROPHYLL METABOLISM). Up to protoporphyrin IX, the biosynthesis of heme and chlorophyll share a common sequence of reactions, and we did not detect any significant changes in the abundance of most of the proteins before that step. By contrast, we found that ferrochelatase (EC:4.99.1.1; Pp1s38_333V6), catalyzing protoheme (heme) generation from protoporphyrin IX, declined at the proteomic level in protoplasts. Chlorophyll biosynthesis proteins were also down-regulated.

We did not detect any significant changes in the abundance of proteins involved in amino acid metabolism, other than for glycine. According to our data, the abundance of some proteins of glycine metabolism was up-regulated in protoplasts (Supplementary Table 7. List 5. GLYCINE, SERINE AND THREONINE METABOLISM).

The abundance of biosynthetic enzymes for chorismate, the key metabolite of the shikimate pathway, increased in protoplasts (Supplementary Table 7; List 6. PHENYLALANINE, TYROSINE AND TRYPTOPHAN BIOSYNTHESIS). Chorismate is the starting point for at least seven metabolic pathways, leading to biosynthesis of aromatic amino acids (phenylalanine, tyrosine, tryptophan) as well as benzoate, salicylate, terpenoid quinones, folate, flavonoids, and a range of other compounds (Tzin and Galili, 2010).

### Comparison of transcriptome and proteome data

In previous research, we performed RNA-seq analysis of the three cell types of moss—protonemata, gametophores, and protoplasts (Fesenko et al., 2015). In this work, we tested for correlation between the RNA-seq data and SWATH-MS data to understand the stress response at the transcriptional and translation levels. We estimated the transcriptional level of 428 SWATH-quantified chloroplast nuclear-coded proteins, 201 of which were differentially accumulated (Supplementary Table 2). We additionally validated the RNA-seq data with RT-qPCR using 10 genes described in this manuscript and randomly representing various functional groups. The expression data from RNA-Seq were largely consistent with those obtained by qRT-PCR (Figure 1D; Supplementary Table 1).

We did not detect any change in transcript accumulation level for the majority of genes, whereas 193 proteins did change in abundance in protoplasts (Supplementary Table 8). In general, we did not detect correlation between the abundance a gene's transcript and that of the corresponding protein (*r* = −0.0027; Figure 2C).

**Figure 4:**
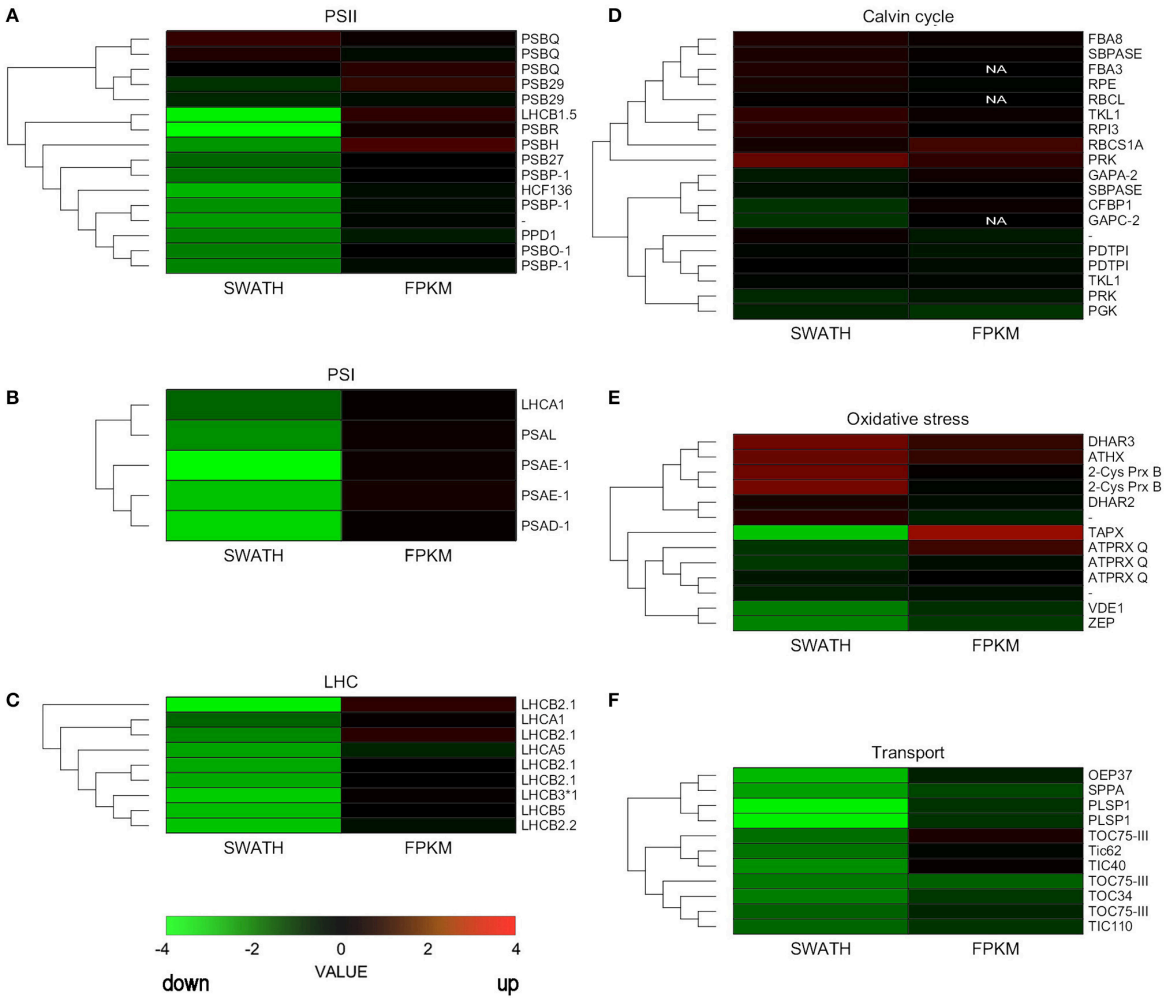
**Hierarchical clustering analysis of transcriptomic and proteomic data**. Heatmap of the gene and protein expression patterns of key genes/proteins in the seven functional groups indicated **(A–F)**. The left portion of each heatmap represents proteomic data (SWATH), and the right represents transcriptomic data (FPKM). The color code is as follows: red indicates up-regulated proteins or transcripts in protoplasts; green indicates down-regulated proteins or transcripts in protoplasts; black indicates unchanged proteins or transcripts in protoplasts. Each row represents the log1,4 (Protoplasts/Protonema. PP/PN) of a protein or the log2 (PP/PN) of a transcript. The color scale of the heatmap ranges from saturated green (value, −4.0) to saturated red (value, 4.0) in the natural logarithmic scale. PSI, Photosystem I; PSII, photosystem II; LHC, light-harvesting complex.

We constructed heatmaps to compare the expression patterns of the quantified proteins and their corresponding transcripts as well as for certain groups of chloroplast proteins such as PSI, PSII, LHC, oxidative stress-related, transport, and ribosomal proteins (Figure 4; Supplementary Figure 2).

Nuclear genes encoding LHC proteins were down-regulated in protoplasts at the proteomic level but slightly increased at the transcriptional level. Similar results were observed for PSI proteins and for most PSII proteins (Figure 4; Supplementary Table 9). However, some genes that encode PSII subunits did not decrease significantly at the transcript or proteomic level.

Some ROS scavenging proteins were induced at the proteomic and transcriptional levels. Others, like zeaxanthin cycle proteins, were down-regulated at the proteomic level. Ribosomal proteins hardly changed at either level, but TIC/TOC complex proteins decreased at both the proteomic and transcriptional levels (Figure 4; Supplementary Table 9).

According to our data, 209 nuclear-encoded chloroplast genes changed neither at the transcriptional level (fold change < 2, *p* < 0.05) nor at the proteomic level (FC < 1.4, *p* < 0.05). Using GO terms, we analyzed the function and localization of the proteins of that group. These proteins were localized in stroma, thylakoids, and chloroplasts membrane and assigned to processes such as biosynthetic process (GO:0009058), response to abiotic stimulus (GO:0009628), single-organism catabolic process (GO:0044712), protein folding (GO:0006457), and cell redox homeostasis (GO:0045454). This latter group of unchanged proteins included proteins from the antioxidant system, like homologs of peroxiredoxin Q (Pp1s95_45V6, Pp1s30_253V6), peroxiredoxin-like protein (Pp1s52_248V6, Pp1s1_230V6), NADPH-dependent thioredoxin reductase C (Pp1s455_2V6), and glutathione reductase (Pp1s13_127V6).

We detected 157 proteins whose abundance was down-regulated in protoplasts but for which there was no change at the level of transcription (Supplementary Table 8). Among these down-regulated proteins, the most significantly decreased were those involved in the light phase of photosynthesis. For example, the proteins of the PSI reaction center, like PSAE1 (Pp1s319_36V6), declined at the proteomic level. The same was true for PSII proteins such as Photosystem II 10 kDa polypeptide, Psb R (Pp1s41_264V6), Photosystem II light harvesting complex (Pp1s76_196V6), Photosystem b protein 33 (Pp1s270_57V6), PsbP-like protein 1 (Pp1s30_39V6), Oxygen-evolving enhancer protein 1-2, PSBO2 (Pp1s306_84V6).

The most interesting group is the one in which the proteins increased in abundance in protoplasts, while the corresponding transcript level remained unchanged. We suppose that these genes are first to react to stress via induction of translation of pre-existing mRNAs. The proteins of this group were involved in the following biological processes: response to stimulus (GO:0050896), oxidation reduction (GO:0055114), cell redox homeostasis (GO:0045454), defense response (GO:0006952). Among them were dehydroascorbate reductase 1 and 2-cys peroxiredoxin B, which play important roles in ROS detoxification. Phosphoribulokinase (Pp1s132_175V6), involved in many different processes including cellular reactions to stress factors, also belonged to this group.

We separately grouped the genes for which the transcript accumulation level decreased but the abundance of the protein either did not change or also decreased (Supplementary Table 8). Members of this group were involved in diverse metabolic processes. For example, abundance was unchanged for such proteins as ADP glucose pyrophosphorylase 2 (Pp1s347_12V6), which regulates starch biosynthesis, and phosphoserine aminotransferase (Pp1s78_211V6), which is important for serine biosynthesis. However, both the level of transcription and abundance of proteins involved in chlorophyll biosynthesis and biosynthesis of other pigments decreased.

For some proteins whose abundance in protoplasts decreased or remained unchanged, the transcriptional level was up-regulated. It is likely that the transcription for these genes is induced at the initial stress phase, which might eventually lead to an increase of their abundance in the proteome. According to biological function, these genes were classified into GO terms including oxylipin biosynthetic process (GO:0031408), response to oxidative stress (GO:0006979), and defense response to bacterium (GO:0009816), among a range of others. For example, one of the main ROS detoxification enzymes, thylakoid ascorbate peroxidase, increased 5.4 times at the transcriptional level but decreased ~3 times at the proteomic level. The Pp1s233_104V6 gene, which is homologous to peroxyredoxin Q of Arabidopsis predicted to be involved in peroxide decomposition using thioredoxin as an electron donor, was induced at the transcriptional level but did not change at the level of the proteome (Perez-Perez et al., 2009). A similar effect was observed for the small subunit of RuBisCo as well as for Rubisco activase.

## Discussion

*P. patens* is resistant to many hostile environment factors and thus is an ideal model for a research on stress resistance mechanisms (Cove et al., 2009; Widiez et al., 2014). However, to date there was no in-depth study of the moss cell proteome at the initial stage of complex, multifactorial stress. Also, little is known about initial stress response in crops (Gong et al., 2015). Using a SWATH-MS approach, we performed label-free comparative quantitative proteomic analysis of chloroplasts isolated from protonemata and protoplasts of the moss *P. patens*. We concentrated on the chloroplast proteome to generate detailed information about the effects of stress conditions on the photosynthetic apparatus of plant cells. We suggest that moss protoplasts should be used as a model for complex stress reaction analysis because the process of protoplast isolation from the cell wall should be similar to drought or salinity stress that leads to plasmolysis, and the treatment with cellulitic enzymes may imitate biotic stress. We integrated the proteomic and transcriptomic data to make a more complete estimation of a cell response to stress factors.

### Protoplast photosynthetic activity

During protoplast isolation, there is a gradual inhibition of photosynthetic activity and quenching of chlorophyll fluorescence (Figures 3A,B). These functional changes correlate with our observed decrease in abundance of almost all ETC components. The main photosystem I and II proteins were all significantly reduced at the protein level, which would lead to inhibition of electron transport reactions in the ETC. It should be emphasized that decline in the quantum yield of PSII (Figure 3B) and, consequently, in the electron transfer rate (Figure 3A), which could be explained by a simple damage of processes of electron transfer or carbon dioxide fixation, is not the only manifestation of the loss of photosynthetic activity. A significant fluorescence quenching is observed in the course of maceration, which can be explained by the rise of non-photochemical quenching coefficient, or directly by the maximum fluorescence value (Fm′) decrease (Figure 3C). In other words, the inhibition of photosynthesis during maceration is not just a passive result of the photosynthetic proteins destruction, but an active response of a cell to maceration, manifested in the development of the intense quenching of excessive excitation in the antenna. It is noteworthy that chlorophyll fluorescence quenching was observed to some extent at lower concentrations of Driselase, which did not lead to complete degradation of cell wall, but induced protein degradation (Figure 3C). This indicates the relation of quenching to protein degradation.

Considering that PSII and LHC are the main chlorophyll-containing complexes in the ETC and that they contribute the most to the chlorophyll fluorescence under physiological conditions, their decreases likely underlies the fluorescence decline that we have observed in this work. According to our data, the abundance of PsbS and LHCSR proteins, which play an important role in non-photochemical quenching (NPQ), was not decreased in *P. patens* protoplasts. Thus, along with a decrease in photosynthetic activity, light-protective photosystem functions remained at the same level, which likely represents a component of the plant's strategy for reaction to stress.

Notably, the observed significant decrease in chlorophyll fluorescence during maceration of protonema is similar to the quenching that occurs in many poikilohydric plants in response to dehydration (Heber et al., 2006; Nabe et al., 2007; Fukuda et al., 2008; Heber, 2008; Fernandez-Marin et al., 2011). Similar quenching can also take place in poikilohydric plant chloroplasts as a response to osmotic stress (Azzabi et al., 2012). Though the quenching mechanism is not well understood, it is known to be triggered by the dehydration-mediated activation of the xanthophyll cycle (Fernandez-Marin et al., 2011), Psbs- and LHCSR-dependent pathways (Peers et al., 2009) and conformational rearrangement of pigment-protein complexes (Heber, 2008). For *P. patens*, there are two simultaneous mechanisms for quenching: Psbs-dependent, which is specific for higher plants, and LHCSR-dependent, which is typical of algae (Gerotto et al., 2012). A specific characteristic of this kind of fluorescence quenching is that it is induced in desiccation-tolerant (DT) plants under stress conditions (dehydration or plasmolysis) regardless of lighting. Importantly, the fluorescence quenching in *P. patens* cells under maceration coincides with what occurs under drought or osmotic stress (Wang et al., 2008). We suppose that these types of stress induce defense reactions that are not just phenomenologically similar but share a common mechanism (at least to a certain degree). It may be that in all the three cases the responses are triggered by a change of interaction between a plasma membrane and a cell wall, namely a partial loss of contact and a change in turgor pressure.

It should be noted that protoplasts of homoiohydric plant *A. thaliana* do not manifest neither decrease in photosynthetic activity nor chlorophyll fluorescence quenching (Riazunnisa et al., 2007). This is in line with our assumption on the specific defensive function of this response inherent to poikilohydric plants.

### Changes in photosynthetic proteins

The plant stress response consists of several phases: initial shock, an acclimation phase, a maintenance phase, an exhaustion phase, and/or a recovery phase (Kosova et al., 2011). In the majority of studies, the changes at the proteomic level under stress conditions have been analyzed after several hours or even days of exposure to stress. Thus, what happens within the initial shock phase of stress is poorly understood (Meng et al., 2014; Gong et al., 2015). It is known that biotic and abiotic stress have negative effects on photosynthesis. For example, under drought in *Xerophyta viscosa* leaves, there is down-regulation of the photosystem II proteins including OEC (oxygen-evolving complex), PsbP, and psbO components (Ingle et al., 2007). Under thermal stress, *Chlamydomonas reinhardtii* down-regulates the abundance of LHC (LHCA5, LHCBM1, and LHCBM3) and OEC (PSBO and PSBP1) components (Muhlhaus et al., 2011). Interestingly, *P. patens* belongs to a group of homoiochlorophyllous organisms that maintain a high level of chlorophyll and protect chloroplasts from destruction under stress. Under stress, homoiochlorophyllous plants protect photosystem proteins from degradation due to destruction of chloroplasts (Georgieva et al., 2007). A number of proteomic studies on the moss reaction to drought, high salinity, and low temperature were conducted with use of 2D-electrophoresis (Wang et al., 2008, 2009). It was shown that under drought or salinity, the abundance of light-harvesting proteins increased and the ATP-synthase subunits were down-regulated, but in general there was no significant damage to the photosynthetic apparatus. At low temperature, the photosystem and light-harvesting proteins were down-regulated and the dark-phase proteins changed bi-directionally (Wang et al., 2009).

**Figure 5:**
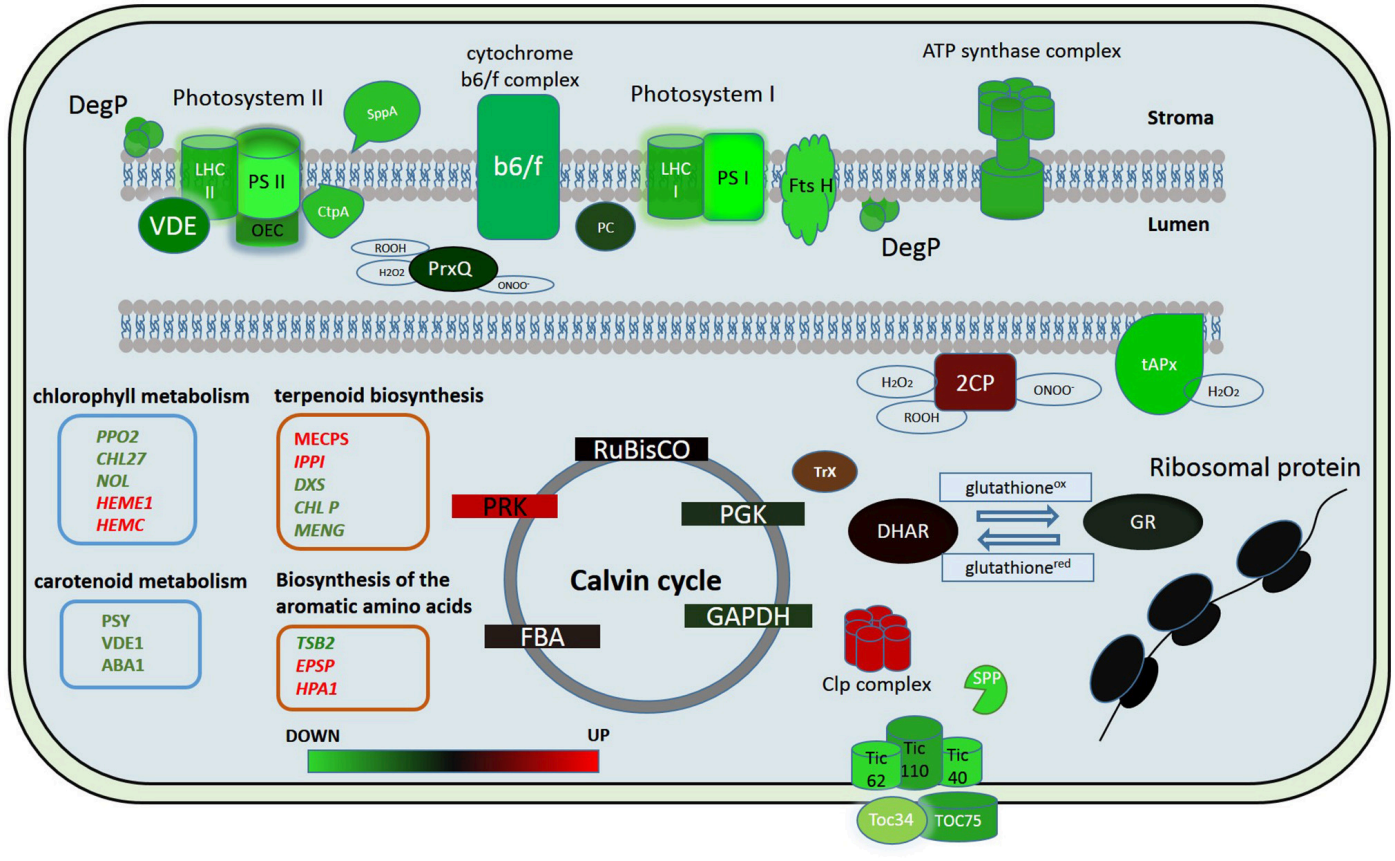
**Putative model of the chloroplast proteome changes in response to short-term complex stress in *P. patens***. The color code is as follows: green indicates down-regulated proteins, red indicates up-regulated proteins. PSI, Photosystem I; PSII, photosystem II; LHC I, light-harvesting complex photosystem I; LHC II, light-harvesting complex photosystem II; OEC, oxygen-evolving complex; PC, plasocyanin; VDE, violaxanthin deepoxidase; Ctp A, Carboxyl-terminal Processing Protease; Prx Q, Peroxiredoxin Q; 2CP, 2-cys-peroxiredoxin; tAPx, thylakoid ascorbate peroxidase tAPX; Trx, thioredoxin; DHAR, dehydroascorbate reductase; GR, glutathione reductase; RuBisCO, Ribulose-1,5-bisphosphate carboxylase/oxygenase; PRK, phosphoribulokinase; PGK, phosphoglycerate kinase; GAPDH, glyceraldehyde-3-phosphate dehydrogenase; FBA, fructose-bisphosphate aldolase; Toc 75, component of the translocon outer membrane (TOC) complex; Toc 34, component of the translocon outer membrane (TOC) complex; TIC40, translocon at the inner envelope membrane of chloroplasts; TIC110, translocon at the inner envelope membrane of chloroplasts; TIC62, translocon at the inner envelope membrane of chloroplasts; SPP, signal peptide peptidase; Clp complex, ATP-dependent Clp protease proteolytic complex.

In our study we showed that the proteins of both cyclic and non-cyclic electron transport were degraded in protoplasts in comparison with protonema cells. We observed the destruction of proteins of photosystems I and II, light-harvesting antenna complexes, oxygen-evolving complex, cytochrome b6/f, and ATP synthase (Figure 5). However, the level of transcription of the corresponding genes remained the same or only slightly decreased. In addition, the abundance of proteins involved in carotenoid biosynthesis was also down-regulated. Accordingly, we suppose that there is degradation of the electron transport chain in protoplasts, likely to prevent ROS accumulation that may damage cells.

Whereas, the degradation of photosystem proteins is not the typical reaction to plasmolysis for the moss, biotic stress can trigger a similar reaction (Bilgin et al., 2010). The components of the oxygen-evolving complex are key factors that modulate ROS actions during immune reactions (Vellosillo et al., 2010). PsbQ degradation results in decreased PSII function, inhibition of the production of protection-associated ROS and depression of the general ability to trigger the transition to pre-programmed cell destruction (Rodriguez-Herva et al., 2012). This fact emphasizes the importance of the oxygen-evolving complex in maintaining thylakoid integrity and immune responses. In our experiments, we observed a decrease in abundance of proteins of this complex in protoplasts.

There are some data on the decrease in abundance of key enzymes of the Calvin cycle under both biotic and abiotic stress (Suzuki et al., 2014). In our study, we did not detect any change in abundance of proteins of Benson-Calvin cycle in the proteome of chloroplasts isolated from protoplasts except for a Phosphoribulokinase (Figure 5). Phosphoribulokinase is a key enzyme of Benson-Calvin cycle that catalyzes phosphorylation of ribulose 5-phosphate (Ru5P) to ribulose 1,5-bisphosphate (RuBP). During the process of photorespiration, ribulose-1,5-bisphosphate serves as a substrate for producing 3-phosphoglycerate (PGA) and 2-phosphoglycolate (2PG or PG). It is known that photorespiration is involved in the defense response, and we suggest that the PRK induction in protoplasts is to produce more ribulose-1,5-bisphosphate for photorespiration. Moreover, the PRK and CP12 protein complex, whose abundance also increases in protoplasts, protects the Calvin cycle enzymes from oxidative stress. Thus, the PRK increase in the chloroplast proteome may also be needed to protect Calvin cycle enzymes during oxidative stress (Marri et al., 2014).

### Chloroplast protein degradation in protoplasts

Though the inhibition of photosynthetic activity and degradation of the chloroplast photosynthetic apparatus occurs along with stress, the mechanisms of these processes have not been thoroughly studied, especially at the proteomic level.

In our study we found that the major part of the chloroplast proteome was decreased in protoplasts. Protein abundance in a proteome depends on transcription, the levels of transcripts, translation and protein degradation (Kristensen et al., 2013). We assume that protein degradation is mainly responsible for protein decreasing in protoplasts. However, we observed that some ribosomal proteins were decreased in protoplasts (Supplementary Table 2). It can point at decreasing of the rate of translation of chloroplast-encoded proteins.

Among DA proteins, we identified several key chloroplast proteases, like FTSH11 (Pp1s9_238V6), FTSH5 (Pp1s44_78V6), FTSH8 (Pp1s118_166V6 and Pp1s156_30V6), SppA (Pp1s25_21V6), and DegP1 (Pp1s160_79V6), whose abundance in the chloroplast proteome decreased 2,18-2,9 times. Interestingly, these proteins are involved in the protein degradation of chloroplast ETC components (Sakamoto, 2006).

Deg proteases are ATP-independent serine endopeptidases involved in biogenesis of photosystem II (PSII) (Schuhmann and Adamska, 2012; Nishimura et al., 2016). Deg 1, Deg5, and Deg8 are lumenal proteases that participate in cleavage of luminal-exposed loops of the D1 protein. Deg1 possesses chaperone and protease activities and has been shown to play important roles in the assembly of PSII dimers and supercomplexes (Jarvi et al., 2015). Deg1 and FtsH proteases act in a cooperative manner and are involved in proteolysis of the thylakoid proteome, including biogenesis of photosystem II (PSII; van Wijk, 2015). The four major isoforms, FtsH 1, −2, −5, and −8 are involved in this process. It has been found that down-regulation of FtsH leads to up-regulation of other chloroplast proteases, including Clp and SppA (Kato et al., 2012). Interestingly, according to our data, Clp protease was up-regulated in protoplasts. Probably, the fast degradation of FtsH proteases in protoplasts leads to up-regulation of Clp protease. The decrease in abundance of the main chloroplast proteases suggests the involvement of other mechanisms in degradation of chloroplast proteins in protoplasts.

In addition to chloroplast proteases, there are three characterized pathways involved in the degradation of chloroplast proteins (Xie et al., 2015). They are degradation pathways via autophagy, senescence associated vacuoles (SAVs), and CV-containing vesicles (CCV; Otegui et al., 2005; Wada et al., 2009; Carrion et al., 2013). Interestingly, we observed decrease in the abundance of thylakoid membrane proteins in protoplasts, but the abundance of proteins of Calvin cycle did not change. It can be explained by activation of different degradation pathways in moss protoplasts. It is known that SAVs and Rubisco-containing bodies (RCBs) are involved in stromal proteins degradation, whereas CV-containing vesicles (CCV) were shown to mediate the vacuolar degradation of stromal proteins, envelope membrane proteins, and thylakoid membrane proteins (Wang and Blumwald, 2014). Stromal and membrane proteins might also be degraded via selective autophagy by their incorporation in ATI-PS bodies (Michaeli et al., 2014). For chloroplast stromal proteins, there are two main pathways of degradation: autophagy and vacuolar transport (senescence-associated vacuoles).

We previously analyzed the endogenous peptides (corresponding to degradation fragments of functionally active proteins) extracted from intact chloroplasts and identified only small amounts of peptides for plastocyanin, cytochrome b559 subunit alpha, and chlorophyll a-b binding protein CP26 (Fesenko et al., 2015). These findings are consistent with the idea of extra-chloroplastic degradation of the majority of the chloroplast proteins in protoplasts. The production of large amounts of peptides of some proteins likely relates to their functions under stress. For example, in our previous study (Fesenko et al., 2015) we showed that a treatment with low concentrations of Driselase that did not cause any visual maceration still resulted in some quenching of fluorescence and triggered the peptidome reaction, namely an increase of chloroplast protein fragments in the cell. It may be that these peptide degradation products of chloroplast proteins are also a part of the stress reaction mechanisms.

### Correlation between the transcription and translation of chloroplast proteins

Over the last years, a great number of studies have been devoted to stress responses and many different methods have been applied to analyze cell transcriptomes (Rasmussen et al., 2013; Beike et al., 2015). We used the data of transcriptional profiling to estimate the correlation between the transcription and translation of genes at the initial phase of stress. The reaction of a plant cell to stress factors at the transcriptomic level does not necessarily reflect the protein level in the proteome and there is no strict correlation between the protein abundance and the level of the corresponding mRNA transcription. Therefore, we integrated the transcriptomic and quantitative proteomic data to elucidate the initial cellular reaction to complex stress. We did not detect any significant correlation between the transcript and protein abundance. The level of transcript accumulation for the majority of proteins identified with SWATH-MS remained unchanged. We conclude that the initial chloroplast reactions to stress relate to changes at the proteomic level.

The fact that the gene transcription and abundance of proteins involved in photosynthetic reactions are independent is also demonstrated in other organisms. The analysis of high intensity light effects on the cyanobacterium *Synechococcus* made it plain that there was a very low correlation between its transcriptome and proteome (Xiong et al., 2015). In many cases, an oppositely directed reaction to light stress was observed: strong induction at the transcriptomic level and insignificant change of the protein abundance in the proteome (Xiong et al., 2015). This phenomenon may depend on changes in protein turnover. We could identify only 11 proteins for which both transcription and translation decreased. The decrease of the level of transcript and protein abundance was specific for genes involved in the biosynthesis chlorophyll and components of the outer chloroplast membrane. According to our data, the first changes at the proteomic level are related to a small number of proteins, for example, ROS detoxification proteins like dihydroascorbate reductase (Pp1s12_401V6), thioredoxin (Pp1s8_193V6), and 2-cys peroxiredoxin B (Pp1s30_345V6). Evidently, these proteins play a key protective role in ROS attack at the initial phase of stress.

In conclusion, we used quantitative proteome analysis (SWATH-MS) and RNA-seq data to analyze the reaction of the chloroplast proteome of the model moss *P. patens* at the initial stage of exposure to complex stress. We discovered that the initial reaction of chloroplast proteome to protoplastation stress is basically the degradation of the light-phase proteins, particularly related to the ETC of chloroplast thylakoids and up-regulation of some ROS detoxifying proteins. Abundance of the dark-phase proteins remains unchanged. Different accumulation of some metabolic enzymes indicates a differences in the regulation of synthesis of the specific metabolites that participate in retrograde signaling. We found no correlation between the level of the gene transcription and protein abundance. We assume that the balance between down- and up-regulation of chloroplast proteome components involved in stress response is rather achieved by changes in transport and translational processes as well as in protein degradation pathways than by alteration of transcription level (Figure 5). The results of this study help to elucidate the dynamics of the chloroplast proteome at the initial phase of stress.

## Author contributions

IF, AS, and VP designed and performed research, analyzed data, and co-wrote the article; GA, AU, and KB contributed new analytic/computational tools and analyzed data; SK, IB, and NA, performed mass-spectrometry experiments; AK, RK, and EP performed research; VG and VI designed research and analyzed data. All authors revised the manuscript critically and approved the final version for publication.

### Conflict of interest statement

The authors declare that the research was conducted in the absence of any commercial or financial relationships that could be construed as a potential conflict of interest.

## References

[B1] Aebersold R. Burlingame A. L. Bradshaw R. A. (2013). Western blots versus selected reaction monitoring assays: time to turn the tables? Mol. Cell. Proteomics 12, 2381–2382. 10.1074/mcp.E113.031658 23756428 PMC3769317

[B2] Asada K. (1999). THE WATER-WATER CYCLE IN CHLOROPLASTS: scavenging of active oxygens and dissipation of excess photons. Annu. Rev. Plant Physiol. Plant Mol. Biol. 50, 601–639. 10.1146/annurev.arplant.50.1.601 15012221

[B3] Atkinson N. J. Lilley C. J. Urwin P. E. (2013). Identification of genes involved in the response of Arabidopsis to simultaneous biotic and abiotic stresses. Plant Physiol. 162, 2028–2041. 10.1104/pp.113.222372 23800991 PMC3729780

[B4] Azzabi G. Pinnola A. Betterle N. Bassi R. Alboresi A. (2012). Enhancement of non-photochemical quenching in the Bryophyte *Physcomitrella patens* during acclimation to salt and osmotic stress. Plant Cell Physiol. 53, 1815–1825. 10.1093/pcp/pcs124 22952250

[B5] Banerjee A. Sharkey T. D. (2014). Methylerythritol 4-phosphate (MEP) pathway metabolic regulation. Nat. Prod. Rep. 31, 1043–1055. 10.1039/c3np70124g 24921065

[B6] Beike A. K. Lang D. Zimmer A. D. Wüst F. Trautmann D. Wiedemann G.. (2015). Insights from the cold transcriptome of *Physcomitrella patens*: global specialization pattern of conserved transcriptional regulators and identification of orphan genes involved in cold acclimation. New Phytol. 205, 869–881. 10.1111/nph.13004 25209349 PMC4301180

[B7] Bilgin D. D. Zavala J. A. Zhu J. Clough S. J. Ort D. R. DeLucia E. H. (2010). Biotic stress globally downregulates photosynthesis genes. Plant Cell Environ. 33, 1597–1613. 10.1111/j.1365-3040.2010.02167.x 20444224

[B8] Burris K. P. Dlugosz E. M. Collins A. G. Stewart C. N. Jr. Lenaghan S. C. (2016). Development of a rapid, low-cost protoplast transfection system for switchgrass (*Panicum virgatum* L.). Plant Cell Rep. 35, 693–704. 10.1007/s00299-015-1913-7 26685665 PMC4757626

[B9] Carrión C. A. Costa M. L. Martínez D. E. Mohr C. Humbeck K. Guiamet J. J. (2013). *In vivo* inhibition of cysteine proteases provides evidence for the involvement of ‘senescence-associated vacuoles’ in chloroplast protein degradation during dark-induced senescence of tobacco leaves. J. Exp. Bot. 64, 4967–4980. 10.1093/jxb/ert285 24106291

[B10] Chaves M. M. Flexas J. Pinheiro C. (2009). Photosynthesis under drought and salt stress: regulation mechanisms from whole plant to cell. Ann. Bot. 103, 551–560. 10.1093/aob/mcn125 18662937 PMC2707345

[B11] Che F. S. Watanabe N. Iwano M. Inokuchi H. Takayama S. Yoshida S.. (2000). Molecular characterization and subcellular localization of protoporphyrinogen oxidase in spinach chloroplasts. Plant Physiol. 124, 59–70. 10.1104/pp.124.1.59 10982422 PMC59122

[B12] Che Y. Fu A. Hou X. McDonald K. Buchanan B. B. Huang W.. (2013). C-terminal processing of reaction center protein D1 is essential for the function and assembly of photosystem II in Arabidopsis. Proc. Natl. Acad. Sci. U.S.A. 110, 16247–16252. 10.1073/pnas.1313894110 24043802 PMC3791762

[B13] Choudhury S. Panda P. Sahoo L. Panda S. K. (2013). Reactive oxygen species signaling in plants under abiotic stress. Plant Signal. Behav. 8:e23681. 10.4161/psb.23681 23425848 PMC7030282

[B14] Cove D. J. Perroud P. F. Charron A. J. McDaniel S. F. Khandelwal A. Quatrano R. S. (2009). The moss *Physcomitrella patens*: a novel model system for plant development and genomic studies. Cold Spring Harb. Protoc. 2009:pdb emo115. 10.1101/pdb.emo115 20147063

[B15] Davey M. R. Anthony P. Power J. B. Lowe K. C. (2005). Plant protoplasts: status and biotechnological perspectives. Biotechnol. Adv. 23, 131–171. 10.1016/j.biotechadv.2004.09.008 15694124

[B16] Doerr A. (2013). Mass spectrometry-based targeted proteomics. Nat. Methods 10, 23. 10.1038/nmeth.2392 23547294

[B17] Estévez J. M. Cantero A. Reindl A. Reichler S. León P. (2001). 1-Deoxy-D-xylulose-5-phosphate synthase, a limiting enzyme for plastidic isoprenoid biosynthesis in plants. J. Biol. Chem. 276, 22901–22909. 10.1074/jbc.M100854200 11264287

[B18] Fernández-Marín B. Míguez F. Becerril J. M. García-Plazaola J. I. (2011). Dehydration-mediated activation of the xanthophyll cycle in darkness: is it related to desiccation tolerance? Planta 234, 579–588. 10.1007/s00425-011-1420-1 21556913

[B19] Fesenko I. A. Arapidi G. P. Skripnikov A. Y. Alexeev D. G. Kostryukova E. S. Manolov A. I.. (2015). Specific pools of endogenous peptides are present in gametophore, protonema, and protoplast cells of the moss *Physcomitrella patens*. BMC Plant Biol. 15:87. 10.1186/s12870-015-0468-7 25848929 PMC4365561

[B20] Foyer C. H. Noctor G. (2011). Ascorbate and glutathione: the heart of the redox hub. Plant Physiol. 155, 2–18. 10.1104/pp.110.167569 21205630 PMC3075780

[B21] Frank W. Ratnadewi D. Reski R. (2005). *Physcomitrella patens* is highly tolerant against drought, salt and osmotic stress. Planta 220, 384–394. 10.1007/s00425-004-1351-1 15322883

[B22] Fu A. He Z. Cho H. S. Lima A. Buchanan B. B. Luan S. (2007). A chloroplast cyclophilin functions in the assembly and maintenance of photosystem II in *Arabidopsis thaliana*. Proc. Natl. Acad. Sci. U.S.A. 104, 15947–15952. 10.1073/pnas.0707851104 17909185 PMC2000425

[B23] Fukuda S. Y. Yamakawa R. Hirai M. Kashino Y. Koike H. Satoh K. (2008). Mechanisms to avoid photoinhibition in a desiccation-tolerant cyanobacterium, Nostoc commune. Plant Cell Physiol. 49, 488–492. 10.1093/pcp/pcn018 18252733

[B24] Genty E. Brazier J. L. Lesca P. Riviere J. L. (1989). Absence of an isotope effect in induction of cytochrome P-450 and xenobiotic metabolizing enzyme activities by stable isotope-labelled phenobarbital isotopomers. Biochem. Pharmacol. 38, 3885–3887. 10.1016/0006-2952(89)90600-X 2597175

[B25] Georgieva K. Szigeti Z. Sarvari E. Gaspar L. Maslenkova L. Peeva V.. (2007). Photosynthetic activity of homoiochlorophyllous desiccation tolerant plant *Haberlea rhodopensis* during dehydration and rehydration. Planta 225, 955–964. 10.1007/s00425-006-0396-8 16983535

[B26] Gerotto C. Alboresi A. Giacometti G. M. Bassi R. Morosinotto T. (2012). Coexistence of plant and algal energy dissipation mechanisms in the moss *Physcomitrella patens*. New Phytol. 196, 763–773. 10.1111/j.1469-8137.2012.04345.x 23005032

[B27] Gillet L. C. Navarro P. Tate S. Röst H. Selevsek N. Reiter L.. (2012). Targeted data extraction of the MS/MS spectra generated by data-independent acquisition: a new concept for consistent and accurate proteome analysis. Mol. Cell. Proteomics 11:O111.016717. 10.1074/mcp.O111.016717 22261725 PMC3433915

[B28] Gong F. Hu X. Wang W. (2015). Proteomic analysis of crop plants under abiotic stress conditions: where to focus our research? Front. Plant Sci. 6:418. 10.3389/fpls.2015.00418 26097486 PMC4456565

[B29] González-Cabanelas D. Wright L. P. Paetz C. Onkokesung N. Gershenzon J. Rodríguez-Concepción M.. (2015). The diversion of 2-C-methyl-D-erythritol-2,4-cyclodiphosphate from the 2-C-methyl-D-erythritol 4-phosphate pathway to hemiterpene glycosides mediates stress responses in *Arabidopsis thaliana*. Plant J. 82, 122–137. 10.1111/tpj.12798 25704332

[B30] Guo J. Morrell-Falvey J. L. Labbé J. L. Muchero W. Kalluri U. C. Tuskan G. A.. (2012). Highly efficient isolation of Populus mesophyll protoplasts and its application in transient expression assays. PLoS ONE 7:e44908. 10.1371/journal.pone.0044908 23028673 PMC3441479

[B31] Heber U. (2008). Photoprotection of green plants: a mechanism of ultra-fast thermal energy dissipation in desiccated lichens. Planta 228, 641–650. 10.1007/s00425-008-0766-5 18587600

[B32] Heber U. Bilger W. Shuvalov V. A. (2006). Thermal energy dissipation in reaction centres and in the antenna of photosystem II protects desiccated poikilohydric mosses against photo-oxidation. J. Exp. Bot. 57, 2993–3006. 10.1093/jxb/erl058 16893979

[B33] Hemmerlin A. Harwood J. L. Bach T. J. (2012). A raison d'etre for two distinct pathways in the early steps of plant isoprenoid biosynthesis? Prog. Lipid Res. 51, 95–148. 10.1016/j.plipres.2011.12.001 22197147

[B34] Hirayama T. Shinozaki K. (2010). Research on plant abiotic stress responses in the post-genome era: past, present and future. Plant J. 61, 1041–1052. 10.1111/j.1365-313X.2010.04124.x 20409277

[B35] Hiss M. Laule O. Meskauskiene R. M. Arif M. A. Decker E. L. Erxleben A.. (2014). Large-scale gene expression profiling data for the model moss *Physcomitrella patens* aid understanding of developmental progression, culture and stress conditions. Plant J. 79, 530–539. 10.1111/tpj.12572 24889180

[B36] Hossain Z. Nouri M. Z. Komatsu S. (2012). Plant cell organelle proteomics in response to abiotic stress. J. Proteome Res. 11, 37–48. 10.1021/pr200863r 22029473

[B37] Hou G. Lou X. Sun Y. Xu S. Zi J. Wang Q.. (2015). Biomarker discovery and verification of esophageal squamous cell carcinoma using integration of SWATH/MRM. J. Proteome Res. 14, 3793–3803. 10.1021/acs.jproteome.5b00438 26224564

[B38] Ingle R. A. Schmidt U. G. Farrant J. M. Thomson J. A. Mundree S. G. (2007). Proteomic analysis of leaf proteins during dehydration of the resurrection plant *Xerophyta viscosa*. Plant Cell Environ. 30, 435–446. 10.1111/j.1365-3040.2006.01631.x 17324230

[B39] Ishihama Y. Oda Y. Tabata T. Sato T. Nagasu T. Rappsilber J.. (2005). Exponentially modified protein abundance index (emPAI) for estimation of absolute protein amount in proteomics by the number of sequenced peptides per protein. Mol. Cell. Proteomics 4, 1265–1272. 10.1074/mcp.M500061-MCP200 15958392

[B40] Järvi S. Suorsa M. Aro E. M. (2015). Photosystem II repair in plant chloroplasts–Regulation, assisting proteins and shared components with photosystem II biogenesis. Biochim. Biophys. Acta 1847, 900–909. 10.1016/j.bbabio.2015.01.006 25615587

[B41] Kangasjärvi S. Neukermans J. Li S. Aro E. M. Noctor G. (2012). Photosynthesis, photorespiration, and light signalling in defence responses. J. Exp. Bot. 63, 1619–1636. 10.1093/jxb/err402 22282535

[B42] Kapri-Pardes E. Naveh L. Adam Z. (2007). The thylakoid lumen protease Deg1 is involved in the repair of photosystem II from photoinhibition in Arabidopsis. Plant Cell 19, 1039–1047. 10.1105/tpc.106.046573 17351117 PMC1867356

[B43] Kato Y. Sun X. Zhang L. Sakamoto W. (2012). Cooperative D1 degradation in the photosystem II repair mediated by chloroplastic proteases in Arabidopsis. Plant Physiol. 159, 1428–1439. 10.1104/pp.112.199042 22698923 PMC3425188

[B44] Kosová K. Vítámvás P. Prášil I. T. Renaut J. (2011). Plant proteome changes under abiotic stress–contribution of proteomics studies to understanding plant stress response. J. Proteomics 74, 1301–1322. 10.1016/j.jprot.2011.02.006 21329772

[B45] Kristensen A. R. Gsponer J. Foster L. J. (2013). Protein synthesis rate is the predominant regulator of protein expression during differentiation. Mol. Syst. Biol. 9, 689. 10.1038/msb.2013.47 24045637 PMC3792347

[B46] Le Bail A. Scholz S. Kost B. (2013). Evaluation of reference genes for RT qPCR analyses of structure-specific and hormone regulated gene expression in *Physcomitrella patens* gametophytes. PLoS ONE 8:e70998. 10.1371/journal.pone.0070998 23951063 PMC3739808

[B47] León I. R. Schwämmle V. Jensen O. N. Sprenger R. R. (2013). Quantitative assessment of in-solution digestion efficiency identifies optimal protocols for unbiased protein analysis. Mol. Cell. Proteomics 12, 2992–3005. 10.1074/mcp.M112.025585 23792921 PMC3790306

[B48] Liu Y.-C. Vidali L. (2011). Efficient polyethylene glycol (PEG) mediated transformation of the moss *Physcomitrella patens*. J. Vis. Exp. 50:2560 10.3791/2560 PMC3169274 21540817

[B49] Marri L. Pesaresi A. Valerio C. Lamba D. Pupillo P. Trost P.. (2010). *In vitro* characterization of Arabidopsis CP12 isoforms reveals common biochemical and molecular properties. J. Plant Physiol. 167, 939–950. 10.1016/j.jplph.2010.02.008 20399532

[B50] Marri L. Thieulin-Pardo G. Lebrun R. Puppo R. Zaffagnini M. Trost P.. (2014). CP12-mediated protection of Calvin-Benson cycle enzymes from oxidative stress. Biochimie 97, 228–237. 10.1016/j.biochi.2013.10.018 24211189

[B51] Meng L. B. Chen Y. B. Lu T. C. Wang Y. F. Qian C. R. Yu Y.. (2014). A systematic proteomic analysis of NaCl-stressed germinating maize seeds. Mol. Biol. Rep. 41, 3431–3443. 10.1007/s11033-014-3205-7 24700167

[B52] Michaeli S. Honig A. Levanony H. Peled-Zehavi H. Galili G. (2014). Arabidopsis ATG8-INTERACTING PROTEIN1 is involved in autophagy-dependent vesicular trafficking of plastid proteins to the vacuole. Plant Cell 26, 4084–4101. 10.1105/tpc.114.129999 25281689 PMC4247578

[B53] Minami A. Nagao M. Ikegami K. Koshiba T. Arakawa K. Fujikawa S.. (2005). Cold acclimation in bryophytes: low-temperature-induced freezing tolerance in *Physcomitrella patens* is associated with increases in expression levels of stress-related genes but not with increase in level of endogenous abscisic acid. Planta 220, 414–423. 10.1007/s00425-004-1361-z 15349781

[B54] Mitra S. Baldwin I. T. (2008). Independently silencing two photosynthetic proteins in *Nicotiana attenuata* has different effects on herbivore resistance. Plant Physiol. 148, 1128–1138. 10.1104/pp.108.124354 18723666 PMC2556805

[B55] Mueller S. J. Lang D. Hoernstein S. N. Lang E. G. Schuessele C. Schmidt A.. (2014). Quantitative analysis of the mitochondrial and plastid proteomes of the moss *Physcomitrella patens* reveals protein macrocompartmentation and microcompartmentation. Plant Physiol. 164, 2081–2095. 10.1104/pp.114.235754 24515833 PMC3982764

[B56] Mühlhaus T. Weiss J. Hemme D. Sommer F. Schroda M. (2011). Quantitative shotgun proteomics using a uniform ^15^N-labeled standard to monitor proteome dynamics in time course experiments reveals new insights into the heat stress response of *Chlamydomonas reinhardtii*. Mol. Cell. Proteomics 10:M110004739. 10.1074/mcp.M110.004739 PMC3186191 21610104

[B57] Nabe H. Funabiki R. Kashino Y. Koike H. Satoh K. (2007). Responses to desiccation stress in bryophytes and an important role of dithiothreitol-insensitive non-photochemical quenching against photoinhibition in dehydrated states. Plant Cell Physiol. 48, 1548–1557. 10.1093/pcp/pcm124 17908696

[B58] Nabity P. D. Zavala J. A. DeLucia E. H. (2009). Indirect suppression of photosynthesis on individual leaves by arthropod herbivory. Ann. Bot. 103, 655–663. 10.1093/aob/mcn127 18660492 PMC2707346

[B59] Nabity P. D. Zavala J. A. DeLucia E. H. (2013). Herbivore induction of jasmonic acid and chemical defences reduce photosynthesis in *Nicotiana attenuata*. J. Exp. Bot. 64, 685–694. 10.1093/jxb/ers364 23264519 PMC3542056

[B60] Nakamura A. Shimada H. Masuda T. Ohta H. Takamiya K. (2001). Two distinct isopentenyl diphosphate isomerases in cytosol and plastid are differentially induced by environmental stresses in tobacco. FEBS Lett. 506, 61–64. 10.1016/S0014-5793(01)02870-8 11591371

[B61] Nishimura K. Kato Y. Sakamoto W. (2016). Chloroplast proteases: updates on proteolysis within and across suborganellar compartments. Plant Physiol. 171, 2280–2293. 10.1104/pp.16.00330 27288365 PMC4972267

[B62] Noguchi S. Shimura G. Kawai M. Suga Y. Samejima H. (1978). Properties of partially purified cellulolytic and plant tissue macerating enzymes of *Irpex lacteus* Fr. in special reference to their application. Agric. Biol. Chem. 42, 339–345.

[B63] Nomura H. Komori T. Uemura S. Kanda Y. Shimotani K. Nakai K.. (2012). Chloroplast-mediated activation of plant immune signalling in Arabidopsis. Nat. Commun. 3:926. 10.1038/ncomms1926 22735454

[B64] Nouri M. Z. Moumeni A. Komatsu S. (2015). Abiotic stresses: insight into gene regulation and protein expression in photosynthetic pathways of plants. Int. J. Mol. Sci. 16, 20392–20416. 10.3390/ijms160920392 26343644 PMC4613210

[B65] Oliver M. J. Velten J. Mishler B. D. (2005). Desiccation tolerance in bryophytes: a reflection of the primitive strategy for plant survival in dehydrating habitats? Integr. Comp. Biol. 45, 788–799. 10.1093/icb/45.5.788 21676830

[B66] Otegui M. S. Noh Y. S. Martinez D. E. Vila Petroff M. G. Staehelin L. A. Amasino R. M.. (2005). Senescence-associated vacuoles with intense proteolytic activity develop in leaves of Arabidopsis and soybean. Plant J. 41, 831–844. 10.1111/j.1365-313X.2005.02346.x 15743448

[B67] Peers G. Truong T. B. Ostendorf E. Busch A. Elrad D. Grossman A. R.. (2009). An ancient light-harvesting protein is critical for the regulation of algal photosynthesis. Nature 462, 518–521. 10.1038/nature08587 19940928

[B68] Pérez-Pérez M. E. Mata-Cabana A. Sánchez-Riego A. M. Lindahl M. Florencio F. J. (2009). A comprehensive analysis of the peroxiredoxin reduction system in the Cyanobacterium *Synechocystis* sp. strain PCC 6803 reveals that all five peroxiredoxins are thioredoxin dependent. J. Bacteriol. 191, 7477–7489. 10.1128/JB.00831-09 19820102 PMC2786602

[B69] Pitzschke A. Persak H. (2012). Poinsettia protoplasts - a simple, robust and efficient system for transient gene expression studies. Plant Methods 8:14. 10.1186/1746-4811-8-14 22559320 PMC3478982

[B70] Polyakov N. B. Slizhikova D. K. Izmalkova M. Y. Cherepanova N. I. Kazakov V. S. Rogova M. A.. (2010). Proteome analysis of chloroplasts from the moss *Physcomitrella patens* (Hedw.) B.S.G. Biochemistry 75, 1470–1483. 10.1134/S0006297910120084 21314618

[B71] Ramegowda V. Senthil-Kumar M. (2015). The interactive effects of simultaneous biotic and abiotic stresses on plants: mechanistic understanding from drought and pathogen combination. J. Plant Physiol. 176, 47–54. 10.1016/j.jplph.2014.11.008 25546584

[B72] Ramel F. Birtic S. Cuiné S. Triantaphylidès C. Ravanat J. L. Havaux M. (2012). Chemical quenching of singlet oxygen by carotenoids in plants. Plant Physiol. 158, 1267–1278. 10.1104/pp.111.182394 22234998 PMC3291260

[B73] Rasmussen S. Barah P. Suarez-Rodriguez M. C. Bressendorff S. Friis P. Costantino P.. (2013). Transcriptome responses to combinations of stresses in Arabidopsis. Plant Physiol. 161, 1783–1794. 10.1104/pp.112.210773 23447525 PMC3613455

[B74] Rensing S. A. Lang D. Zimmer A. D. Terry A. Salamov A. Shapiro H.. (2008). The Physcomitrella genome reveals evolutionary insights into the conquest of land by plants. Science 319, 64–69. 10.1126/science.1150646 18079367

[B75] Riazunnisa K. Padmavathi L. Scheibe R. Raghavendra A. S. (2007). Preparation of Arabidopsis mesophyll protoplasts with high rates of photosynthesis. Physiol. Plant. 129, 879–886. 10.1111/j.1399-3054.2007.00867.x

[B76] Robinson M. D. McCarthy D. J. Smyth G. K. (2010). edgeR: a Bioconductor package for differential expression analysis of digital gene expression data. Bioinformatics 26, 139–140. 10.1093/bioinformatics/btp616 19910308 PMC2796818

[B77] Rodríguez-Herva J. J. Gonzélez-Melendi P. Cuartas-Lanza R. Antúnez-Lamas M. Río-Alvarez I. Li Z.. (2012). A bacterial cysteine protease effector protein interferes with photosynthesis to suppress plant innate immune responses. Cell. Microbiol. 14, 669–681. 10.1111/j.1462-5822.2012.01749.x 22233353

[B78] Sakamoto W. (2006). Protein degradation machineries in plastids. Annu. Rev. Plant Biol. 57, 599–621. 10.1146/annurev.arplant.57.032905.105401 16669775

[B79] Salvucci M. E. Crafts-Brandner S. J. (2004a). Inhibition of photosynthesis by heat stress: the activation state of Rubisco as a limiting factor in photosynthesis. Physiol. Plant. 120, 179–186. 10.1111/j.0031-9317.2004.0173.x 15032851

[B80] Salvucci M. E. Crafts-Brandner S. J. (2004b). Relationship between the heat tolerance of photosynthesis and the thermal stability of rubisco activase in plants from contrasting thermal environments. Plant Physiol. 134, 1460–1470. 10.1104/pp.103.038323 15084731 PMC419822

[B81] Schuhmann H. Adamska I. (2012). Deg proteases and their role in protein quality control and processing in different subcellular compartments of the plant cell. Physiol. Plant. 145, 224–234. 10.1111/j.1399-3054.2011.01533.x 22008015

[B82] Suzuki N. Rivero R. M. Shulaev V. Blumwald E. Mittler R. (2014). Abiotic and biotic stress combinations. New Phytol. 203, 32–43. 10.1111/nph.12797 24720847

[B83] Tanaka R. Oster U. Kruse E. Rudiger W. Grimm B. (1999). Reduced activity of geranylgeranyl reductase leads to loss of chlorophyll and tocopherol and to partially geranylgeranylated chlorophyll in transgenic tobacco plants expressing antisense RNA for geranylgeranyl reductase. Plant Physiol. 120, 695–704. 10.1104/pp.120.3.695 10398704 PMC59307

[B84] Thimm O. Bläsing O. Gibon Y. Nagel A. Meyer S. Krüger P.. (2004). MAPMAN: a user-driven tool to display genomics data sets onto diagrams of metabolic pathways and other biological processes. Plant J. 37, 914–939. 10.1111/j.1365-313X.2004.02016.x 14996223

[B85] Tiew T. W. Sheahan M. B. Rose R. J. (2015). Peroxisomes contribute to reactive oxygen species homeostasis and cell division induction in Arabidopsis protoplasts. Front. Plant Sci. 6:658. 10.3389/fpls.2015.00658 26379686 PMC4549554

[B86] Toprak U. H. Gillet L. C. Maiolica A. Navarro P. Leitner A. Aebersold R. (2014). Conserved peptide fragmentation as a benchmarking tool for mass spectrometers and a discriminating feature for targeted proteomics. Mol. Cell. Proteomics 13, 2056–2071. 10.1074/mcp.O113.036475 24623587 PMC4125737

[B87] Trapnell C. Williams B. A. Pertea G. Mortazavi A. Kwan G. van Baren M. J.. (2010). Transcript assembly and quantification by RNA-Seq reveals unannotated transcripts and isoform switching during cell differentiation. Nat. Biotechnol. 28, 511–515. 10.1038/nbt.1621 20436464 PMC3146043

[B88] Trotta A. Rahikainen M. Konert G. Finazzi G. Kangasjärvi S. (2014). Signalling crosstalk in light stress and immune reactions in plants. Philos. Trans. R. Soc. Lond. B Biol. Sci. 369:20130235. 10.1098/rstb.2013.0235 24591720 PMC3949398

[B89] Tzin V. Galili G. (2010). The biosynthetic pathways for shikimate and aromatic amino acids in *Arabidopsis thaliana*. Arabidopsis Book 8:e0132. 10.1199/tab.0132 22303258 PMC3244902

[B90] van Wijk K. J. (2015). Protein maturation and proteolysis in plant plastids, mitochondria, and peroxisomes. Annu. Rev. Plant Biol. 66, 75–111. 10.1146/annurev-arplant-043014-115547 25580835

[B91] Vass I. (2012). Molecular mechanisms of photodamage in the Photosystem II complex. Biochim. Biophys. Acta 1817, 209–217. 10.1016/j.bbabio.2011.04.014 21565163

[B92] Vellosillo T. Vicente J. Kulasekaran S. Hamberg M. Castresana C. (2010). Emerging complexity in reactive oxygen species production and signaling during the response of plants to pathogens. Plant Physiol. 154, 444–448. 10.1104/pp.110.161273 20921160 PMC2948990

[B93] Vizcaíno J. A. Deutsch E. W. Wang R. Csordas A. Reisinger F. Ríos D.. (2014). ProteomeXchange provides globally coordinated proteomics data submission and dissemination. Nat. Biotechnol. 32, 223–226. 10.1038/nbt.2839 24727771 PMC3986813

[B94] Vranová E. Coman D. Gruissem W. (2013). Network analysis of the MVA and MEP pathways for isoprenoid synthesis. Annu. Rev. Plant Biol. 64, 665–700. 10.1146/annurev-arplant-050312-120116 23451776

[B95] Wada S. Ishida H. Izumi M. Yoshimoto K. Ohsumi Y. Mae T.. (2009). Autophagy plays a role in chloroplast degradation during senescence in individually darkened leaves. Plant Physiol. 149, 885–893. 10.1104/pp.108.130013 19074627 PMC2633819

[B96] Wang L. Liang W. Xing J. Tan F. Chen Y. Huang L.. (2013). Dynamics of chloroplast proteome in salt-stressed mangrove *Kandelia candel* (L.) Druce. J. Proteome Res. 12, 5124–5136. 10.1021/pr4006469 24070322

[B97] Wang S. Blumwald E. (2014). Stress-induced chloroplast degradation in Arabidopsis is regulated via a process independent of autophagy and senescence-associated vacuoles. Plant Cell 26, 4875–4888. 10.1105/tpc.114.133116 25538186 PMC4311210

[B98] Wang X. Yang P. Gao Q. Liu X. Kuang T. Shen S.. (2008). Proteomic analysis of the response to high-salinity stress in *Physcomitrella patens*. Planta 228, 167–177. 10.1007/s00425-008-0727-z 18351383

[B99] Wang X. Yang P. Zhang X. Xu Y. Kuang T. Shen S.. (2009). Proteomic analysis of the cold stress response in the moss, *Physcomitrella patens*. Proteomics 9, 4529–4538. 10.1002/pmic.200900062 19670371

[B100] Widiez T. Symeonidi A. Luo C. Lam E. Lawton M. Rensing S. A. (2014). The chromatin landscape of the moss *Physcomitrella patens* and its dynamics during development and drought stress. Plant J. 79, 67–81. 10.1111/tpj.12542 24779858

[B101] Xiao L. Zhang L. Yang G. Zhu H. He Y. (2012). Transcriptome of protoplasts reprogrammed into stem cells in *Physcomitrella patens*. PLoS ONE 7:e35961. 10.1371/journal.pone.0035961 22545152 PMC3335808

[B102] Xiao Y. Savchenko T. Baidoo E. E. Chehab W. E. Hayden D. M. Tolstikov V.. (2012). Retrograde signaling by the plastidial metabolite MEcPP regulates expression of nuclear stress-response genes. Cell 149, 1525–1535. 10.1016/j.cell.2012.04.038 22726439

[B103] Xie Q. Michaeli S. Peled-Zehavi H. Galili G. (2015). Chloroplast degradation: one organelle, multiple degradation pathways. Trends Plant Sci. 20, 264–265. 10.1016/j.tplants.2015.03.013 25865278

[B104] Xiong Q. Feng J. Li S. T. Zhang G. Y. Qiao Z. X. Chen Z.. (2015). Integrated transcriptomic and proteomic analysis of the global response of Synechococcus to high light stress. Mol. Cell. Proteomics 14, 1038–1053. 10.1074/mcp.M114.046003 25681118 PMC4390250

[B105] You J. Chan Z. (2015). ROS regulation during abiotic stress responses in crop plants. Front. Plant Sci. 6:1092. 10.3389/fpls.2015.01092 26697045 PMC4672674

[B106] Zhang Y. Su J. Duan S. Ao Y. Dai J. Liu J.. (2011). A highly efficient rice green tissue protoplast system for transient gene expression and studying light/chloroplast-related processes. Plant Methods 7:30. 10.1186/1746-4811-7-30 21961694 PMC3203094

[B107] Zimmer A. D. Lang D. Buchta K. Rombauts S. Nishiyama T. Hasebe M.. (2013). Reannotation and extended community resources for the genome of the non-seed plant *Physcomitrella patens* provide insights into the evolution of plant gene structures and functions. BMC Genomics 14:498. 10.1186/1471-2164-14-498 23879659 PMC3729371

