# Supplementary material for: The Physcomitrella patens Chloroplast Proteome Changes in Response to Protoplastation
Source: Front Plant Sci. 2016 Nov 4;7:1661. doi: 10.3389/fpls.2016.01661 (PMC5095126; doi:10.3389/fpls.2016.01661)
Supplement: Supplementary file 8 [file Table8.PDF]

The *Physcomitrella patens* chloroplast proteome changes in response to protoplastation

Igor Fesenko[1]*, Anna Seredina[1], Georgij Arapidi[1]

Correspondence: Igor Fesenko, fesigor@gmail.com

Supplementary Table 8. Comparative analysis of RNA-seq and SWATH-MS data

		Protein		
		Unchanged	Decrease in protoplasts	Increase in protoplasts
**Transcript**	Unchanged	**209 genes**<br>GO Biological Process:<br>biosynthetic process, nitrogen compound metabolic process	**157 genes**<br>GO Biological Process:<br>metabolic process, oxidation-reduction process, cofactor metabolic process	**22 genes**<br>GO Biological Process:<br>small molecule metabolic process, generation of precursor metabolites and energy, catabolic process
	Decrease in protoplasts	**15 genes**<br>GO Biological Process:<br>nitrogen compound metabolic process, carboxylic acid metabolic process	**9 genes**<br>GO Biological Process:<br>organic cyclic compound biosynthetic process, pigment metabolic process	**0**
	Increase in protoplasts	**11 genes**<br>GO Biological Process:<br>organic acid metabolic process	**5 genes**<br>GO Biological Process:<br>oxidation-reduction process, oxylipin biosynthetic process	**0**
