# Supplementary material for: The Physcomitrella patens Chloroplast Proteome Changes in Response to Protoplastation
Source: Front Plant Sci. 2016 Nov 4;7:1661. doi: 10.3389/fpls.2016.01661 (PMC5095126; doi:10.3389/fpls.2016.01661)
Supplement: Supplementary file 10 [file Image1.PDF]

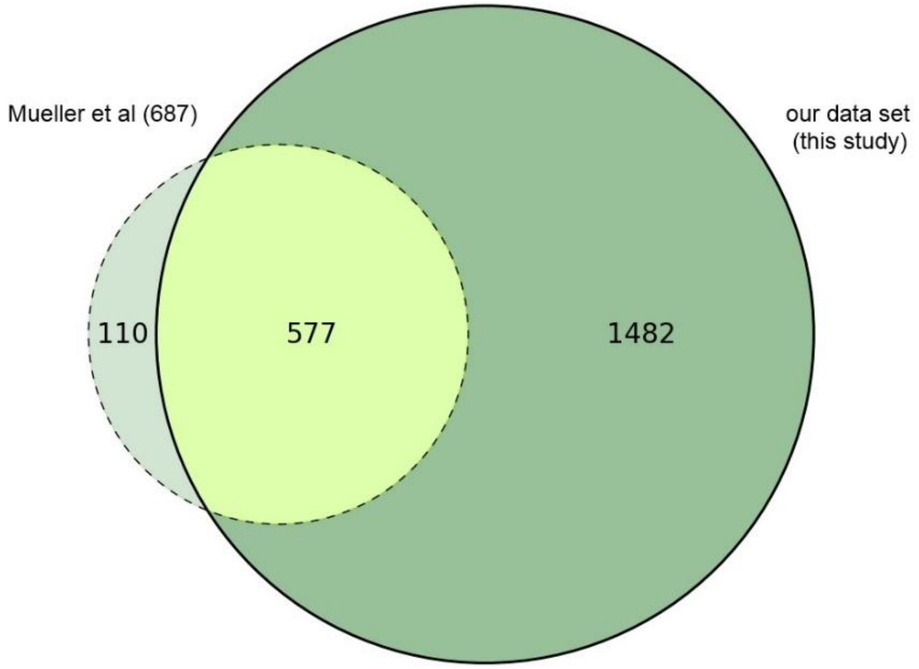

Supplementary Figure 1. Venn diagram for the comparison of proteins identified in this study and the previously published results (Mueller et al, 2014).
