# Supplementary material for: The Physcomitrella patens Chloroplast Proteome Changes in Response to Protoplastation
Source: Front Plant Sci. 2016 Nov 4;7:1661. doi: 10.3389/fpls.2016.01661 (PMC5095126; doi:10.3389/fpls.2016.01661)
Supplement: Supplementary file 11 [file Image2.PDF]

The *Physcomitrella patens* chloroplast proteome changes in response to protoplastation

Igor Fesenko[1]*, Anna Seredina[1], Georgij Arapidi[1]

Correspondence: Igor Fesenko, fesigor@gmail.com

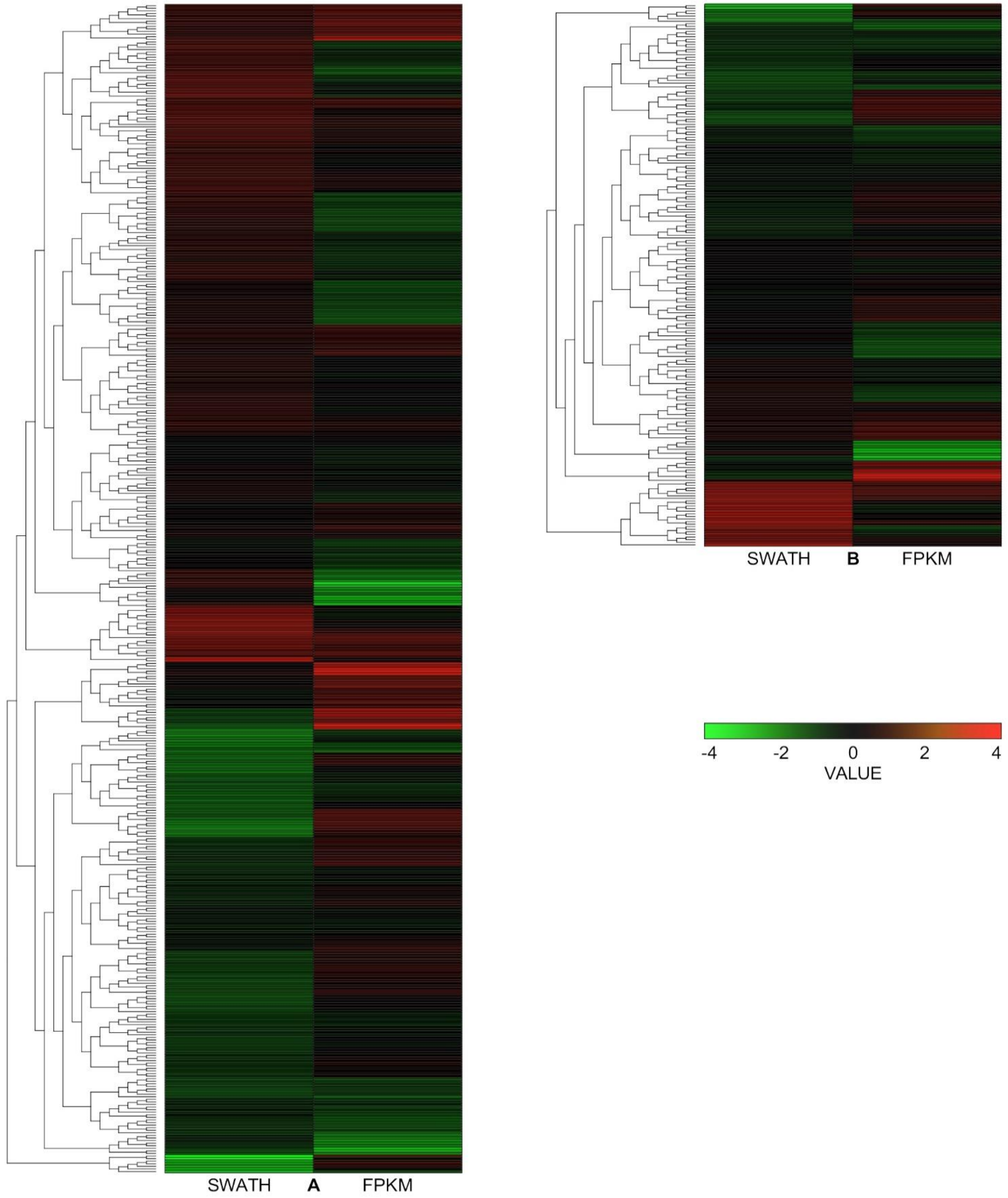

Supplementary Figure 2. Heatmap of all the SWATH quantified proteins and their corresponding mRNAs (A), Heatmap of DEPs and their corresponding mRNAs (B).
